# Impact of the female and hermaphrodite forms of *Opuntia robusta* on the plant defence hypothesis

**DOI:** 10.1038/s41598-021-91524-5

**Published:** 2021-06-08

**Authors:** Mariusz Krzysztof Janczur, Emilio González-Camarena, Hector Javier León-Solano, Mario Alberto Sandoval-Molina, Bartosz Jenner

**Affiliations:** 1 Research Group in Ecology and Evolutionary Biology, Department of Natural Sciences, Autonomous University of the State of Mexico, México, Carretera Toluca-Tlachaloya, km 18, Cerrillo Piedras Blancas, 50200 Toluca, Estado de México México; 2 Graduate Program in Agricultural Sciences and Natural Resources (PCARN), Autonomous University of the State of Mexico, México, Carretera Toluca-Tlachaloya, km 18, Cerrillo Piedras Blancas, 50200 Toluca, Estado de México México; 3 Instituto de Ecología A.C., Red de Ecología Funcional. Carretera Antigua a Coatepec, 351, El Haya, 91070 Xalapa, Veracruz México; 4 Actelion Pharmaceuticals Ltd, Gewerbestrasse 16, 4123 Allschwil, Switzerland

**Keywords:** Evolutionary ecology, Secondary metabolism

## Abstract

The optimal defence hypothesis predicts that increased plant defence capabilities, lower levels of damage, and lower investment in vegetative biomass will occur more frequently in sexual forms with higher resource-demanding tissue production and/or younger plant parts. We aimed to examine the effects of sexual form, cladode, and flower age on growth rate, herbivore damage, and 4-hydroxybenzoic acid (4-HBA), chlorogenic acid, and quercetin (QUE) concentrations in *Opuntia robusta* plants in central Mexico. Our findings demonstrated that hermaphrodite flowers showed faster growth and lesser damage than female flowers. The effect of cladode sexual forms on 4-HBA and QUE occurrence was consistent with the predictions of the optimal defence hypothesis. However, chlorogenic acid occurrences were not significantly affected by sexual forms. Old cladodes exhibited higher QUE and 4-HBA occurrences than young cladodes, and hermaphrodites exhibited higher 4-HBA concentrations than females. Resource allocation for reproduction and secondary metabolite production, and growth was higher and lower, respectively, in females, compared to hermaphrodites, indicating a trade-off between investment in reproduction, growth, and secondary metabolite production. Secondary metabolite concentrations in *O. robusta* plants were not negatively correlated with herbivore damage, and the two traits were not accurate predictors of plant reproductive output.

## Introduction

Several hypotheses have attempted to identify evolutionary patterns relating to the physiological costs and plant defence strategies against herbivores. According to the optimal defence hypothesis, chemical defences are allocated as a function of tissues’ value in terms of fitness. Therefore, the concept of cost is related to plant tissue age, because younger tissues have higher assimilation rates than older tissues, and to different sexual forms of plants, because female tissues are expected to be more resource-demanding than male or hermaphrodite tissues^1^. When extending this hypothesis to plants with different sexual forms, it is predicted that more resource-demanding sexual forms will be better defended than less resource-demanding forms, because a reduction in their fitness due to tissue loss is greater than that due to a lower allocation of resources for defence^2,3^. Consequently, younger tissues or plants should be better defended than older ones, because of their higher photosynthetic potential^4^. Plants that exhibit several sexual forms are ideal specimens for testing the validity of the optimal defence hypothesis, as the cost of the reproductive tissues is different for each sex^2^. Most predictions of the optimal defence hypothesis regarding plant sex have been confirmed in a meta-analysis by Cornelissen and Stiling^5^.

Few studies have shown that resource allocation to reproduction is higher in female and hermaphrodite plants, as compared to males, which represent a less costly sexual form^1^. Moreover, according to Coley’s resource availability hypothesis, defence investment is related to the relative growth rate of a plant species, which is associated with the habitat quality. Therefore, slow-growing species or individuals allocate more resources to defence than faster-growing species or individuals^6^. This phenomenon has been confirmed in *Betula* spp., *Salix* spp., *Populus* spp.^7^, and *Acer negundo*^8^.

The predictions of the resource availability and optimal defence hypotheses are compatible with regard to the cost of tissues, because, according to the former, the biomass of plants growing in less productive environments is more valuable in terms of energy and fitness, because their growth is slower, and according to the latter, the recovery of more costly tissues is more difficult, due to their lower assimilation rate and the greater negative effects associated with their loss on fitness. Both hypotheses predict that more valuable (slower growing) tissues will be better defended. However, the resource competition hypothesis states that because female plants invest more resources in reproduction than male or hermaphrodite plants, their growth is slower, and they invest less energy in defence, exhibit lower secondary metabolites levels, and consequently have higher herbivory levels^9^.

To protect themselves against herbivores, pathogens, and abiotic stress, plants produce substances frequently referred to as ‘secondary metabolites’. These metabolites were previously considered to be plant metabolic waste; however, more recent studies have shown that they are key components of active and potent defence mechanisms of plants against predators^10^. Secondary metabolites are produced not only in response to herbivores, but also in response to abiotic stress factors, such as UV radiation, temperature, humidity, and precipitation. However, no congruent theory has explained the production of secondary metabolites in response to both biotic and abiotic factors^11^. This may be because the array of secondary metabolites produced by plants in response to stress factors is large, and several secondary metabolites share common precursors^10^. Therefore, the simultaneous production of secondary metabolites in response to both biotic and abiotic factors is associated with several trade-offs, as a response towards a biotic stress factor may imply lower responses towards an abiotic factor, and vice versa. Studies have shown that herbivore-induced secondary metabolite production can be suppressed in certain species under a range of abiotic conditions^12,13^. Such trade-offs will be more evident in sexual forms with more costly tissues, because a higher investment of resources in vegetative tissues implies a higher extent of defence, as predicted by the optimal defence and resource availability hypotheses, or because a higher investment of resources in more costly tissues channelises more energy from the energy reservoir maintained for defence, as predicted by the resource competition hypothesis. A comparison of the extent of secondary metabolite production induced by exposure to different abiotic stresses at different intensity levels will provide insight into the differences in cost for different plant sexual forms. If abiotic factors associated with plant productivity (global radiation, potential evapotranspiration, and relative humidity) have a significant effect on one secondary metabolite, and have an opposite effect on another, it will provide evidence of their cost, and will suggest that there is a trade-off of resources between both secondary metabolites. Analogously, if such a trade-off exists in one sexual form but not in the other, it will indicate that the former is more resource-demanding. In addition to a slower growth rate, this would be evidence of a higher relative vegetative tissue cost.

There exist a knowledge gap concerning the intersexual differences in the extent of secondary metabolites production in plant with different sexual forms. A few studies claim that female plants produce higher concentrations of secondary metabolites than male plants^14,15^ and only one study shows that male plants produce higher concentrations of secondary metabolites than female plants^16^. There is no study concerning such differences between unisexual and hermaphrodite plants.

*Opuntia robusta* Wendl. (Cactaceae) is an ideal species for testing the optimal defence hypothesis, the resource availability hypothesis, and the resource competition hypothesis, as it has three sexual forms, namely male, female, and hermaphrodite. Furthermore, it is easy to distinguish between the age classes of its cladodes.

Although studies on secondary metabolite concentrations in *O. robusta* cladodes are few^17,18^, the defensive functions of **chlorogenic acid** (CGA)^19–21^, **4-hydroxybenzoic acid** (4-HBA)^22^, and **quercetin** (QUE)^23,24^ have been confirmed in other plants. Studies have shown that **salicylic acid** (SA; 2**-**hydroxybenzoic acid) is involved in plant defence^25,26^; however, the possible inductive function of this metabolite in response to herbivory in the *Opuntia* genus remains elusive (Supplementary Information).

Based on the optimal defence hypothesis, we predict that the concentrations and occurrence of different secondary metabolites in female cladodes of *O. robusta* will be higher than those in hermaphrodite cladodes, and that younger cladodes of both female and hermaphrodite forms will contain higher concentrations of secondary metabolites than older cladodes. In a previous study, we observed a trade-off between the phenol contents of parental and daughter cladodes; therefore, we predict that the concentrations of secondary metabolites will be higher in daughter cladodes than those in parental cladodes. We expect the existence of trade-off between the substances, 4-HBA, CGA, QUE, and SA, studied in the plant tissue, because CGA is metabolised directly from shikimic acid, whereas QUE, SA, and 4-HBA are metabolised from shikimic acid via the phenylalanine-cinnamic acid route^10^, even though synthesis via alternate pathways is possible^27^. If the amount of common substrate (shikimic acid) is limited, an increase in the concentration of one of these metabolites should lead to a decrease in the concentration of the others.

Our limited understanding of secondary metabolite production costs in plants^28^, and the knowledge of the existence of trade-offs between investments in the production of different secondary metabolites^12,13^, suggest that traditional hypotheses explaining the mechanisms by which selective pressures shape plant responses to biotic stress factors may be intrinsically incorrect. We intend to answer the following questions: 1) are tissues of female individuals more costly than those of hermaphrodite individuals?; 2) is the concentration/occurrence of secondary metabolites higher in female than that in hermaphrodite individuals?; 3) are female individuals less prone to herbivore damage than hermaphrodite individuals?; 4) is the concentration and occurrence of secondary metabolites higher in younger tissues than that in older vegetative tissues?; 5) is there evidence of trade-offs between investment in defence, growth, and reproduction?; 6) are female vegetative size estimators lower than those of hermaphrodites?; 7) does the existence of trade-offs between different secondary metabolites affect the predictions of the plant defence hypothesis? To the best of our knowledge, this study is the first to examine the implications of the existence of separate sexual forms of *O. robusta* on the plant defence hypothesis.

## Results

### Are tissues of female individuals costlier than those of hermaphrodite individuals?

**Figure 1:**
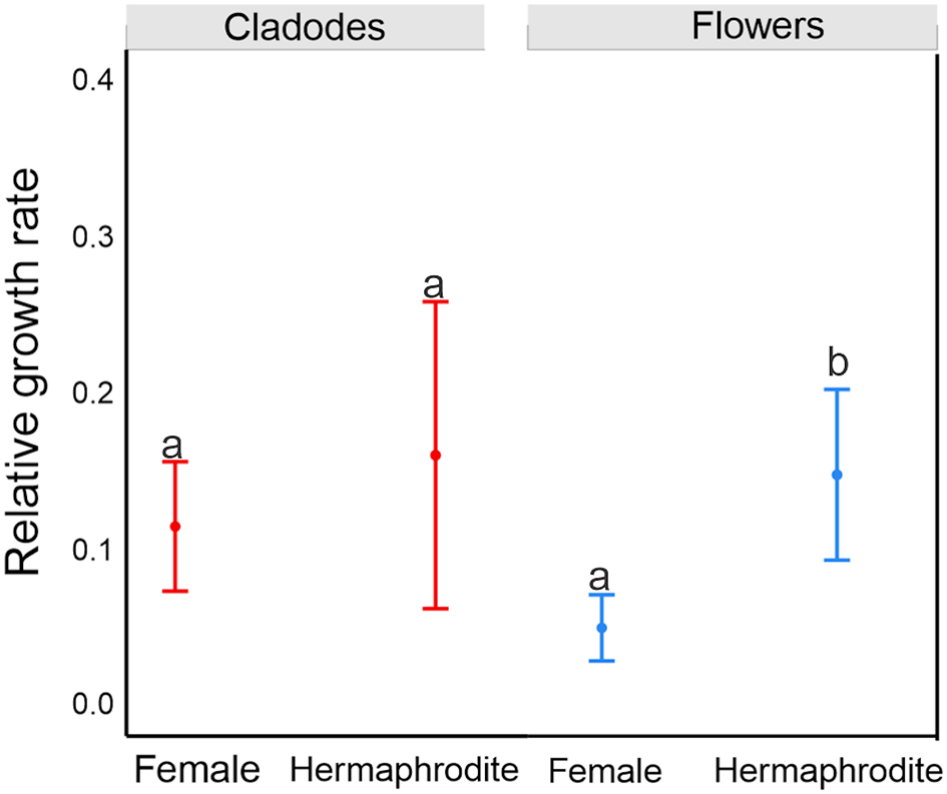
Effects of the sexual form (female, hermaphrodite) and structure (cladode and flower buds) of *Opuntia robusta* on relative growth rates of plant structures. The figure represents the estimated mean values and confidence intervals obtained from a GLM, with Gamma error distribution and a log link function. The explanatory variables were sex, type of structure, their interactions, and cumulative herbivory. The difference between female and hermaphrodite flowers was significant (*P* ≤ 0.001).

The average relative growth rate of cladodes was not significantly affected by sexual forms at *P* ≤ 0.05. However, hermaphrodite flowers had significantly higher relative growth rates than female flowers. Furthermore, the growth rates of the two sexual forms were not significantly affected by herbivory at *P* ≤ 0.05  (Fig. 1; Table S1; Dataset in Sandoval and Janczur^29^ online). The present and previous studies performed by us using this population of *O. robusta* showed a consistently higher tissue growth rate in hermaphrodites than in female individuals over the years^30^.

**Figure 2:**
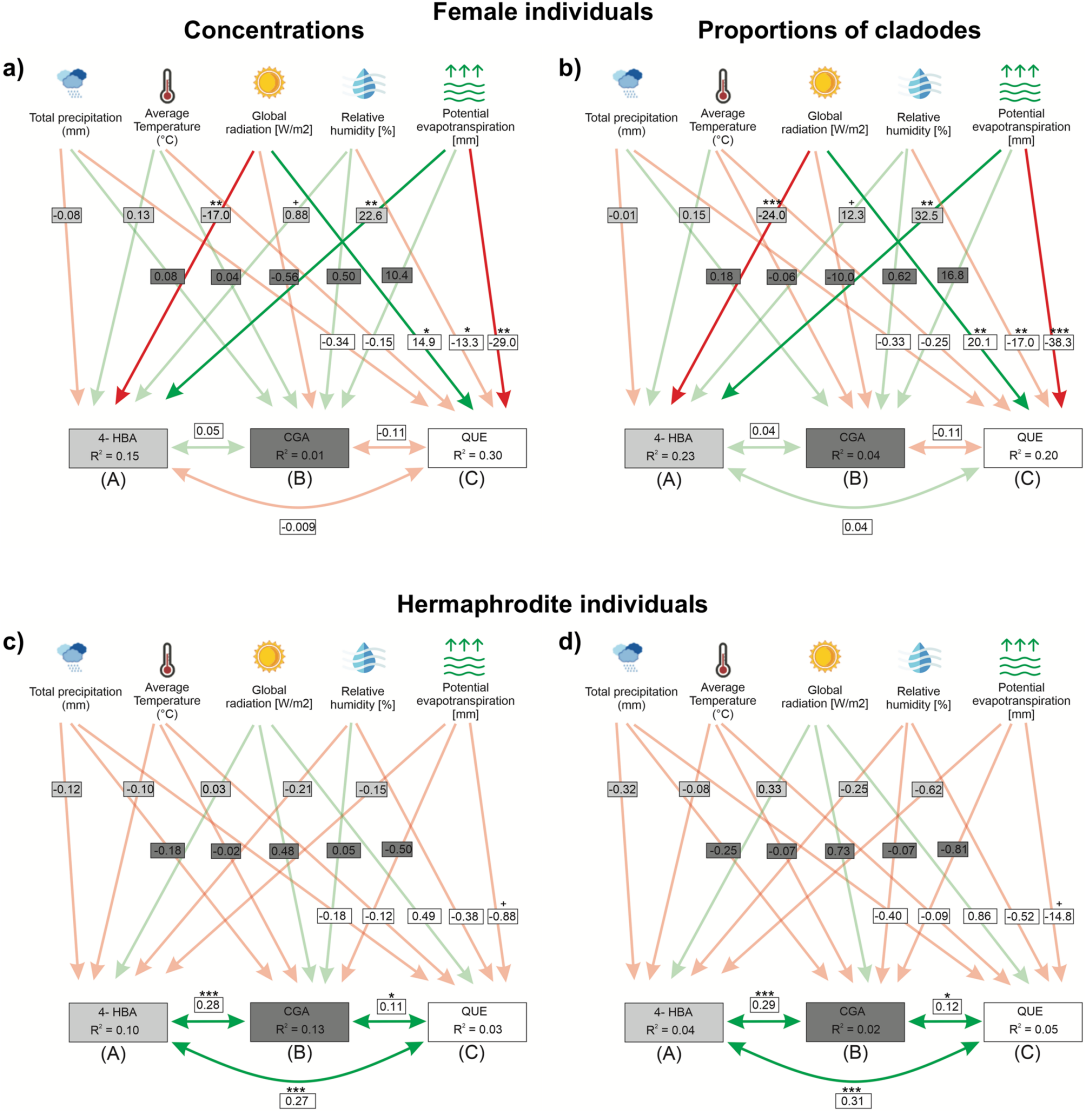
Structural equation model (SEM) results showing the relationship between the environmental variables and the concentration (**a**) and occurrence (**b**) of 4-hydroxybenzoic acid (4-HBA), chlorogenic acid (CGA), and quercetin (QUE) in female individuals, and the concentration (**c**) and occurrence (**d**) of these metabolites in hermaphrodite *Opuntia robusta* plants. For each figure, section (**A**) corresponds to the effects of the environment on the concentration of 4-HBA, (**B**) corresponds to the effects of the environment on the concentration of CGA, and (**C**) corresponds to the effects of the environment on the concentration of QUE. Green lines represent positive effects, while red lines indicate negative effects. Numbers inside the boxes on the arrows represent standardized path coefficients. R^2^ for each component in the model is given inside the box. Solid lines (intense colour) represent significant pathways. Significance: + – *P* slightly higher than 0.05, *p < 0.05, ** *p* < 0.01, and *** *p* < 0.001. The model for female individuals was well supported by the data (Fisher’s C = 3.71; *P* = 0.71), but the model for hermaphrodite individuals was not (Fisher’s C = 67.34; *P* < 0.05).

In females, 4-HBA and QUE concentrations were significantly negatively and positively affected, respectively, by global radiation. However, potential evapotranspiration had significant positive and negative effects on 4-HBA and QUE concentrations, respectively. Humidity also had positive and negative effects on the 4-HBA and QUE, respectively. (Fig. 2a). The effect of potential evapotranspiration, global radiation and relative humidity on the proportion of cladodes harbouring 4-HBA and QUE were similar to that on the concentrations of these metabolites (Fig. 2b). While QUE, 4-HBA, and CGA concentrations in hermaphrodites were negatively or positively affected by meteorological parameters, the effects were not significant at *P* ≤ 0.05 (Fig. 2c). The pattern was the same for the proportion of cladodes harbouring these three metabolites and the relationship between them. The same analysis showed a positive and significant relationship between both the concentrations and occurrence of these three metabolites (Fig. 2d; Table S2 online^31^). The monthly dynamics of the meteorological variables and the correlations between each pair are described in the Supplementary Information file (Results S1, Table S2, Figure S1).

**Figure 3:**
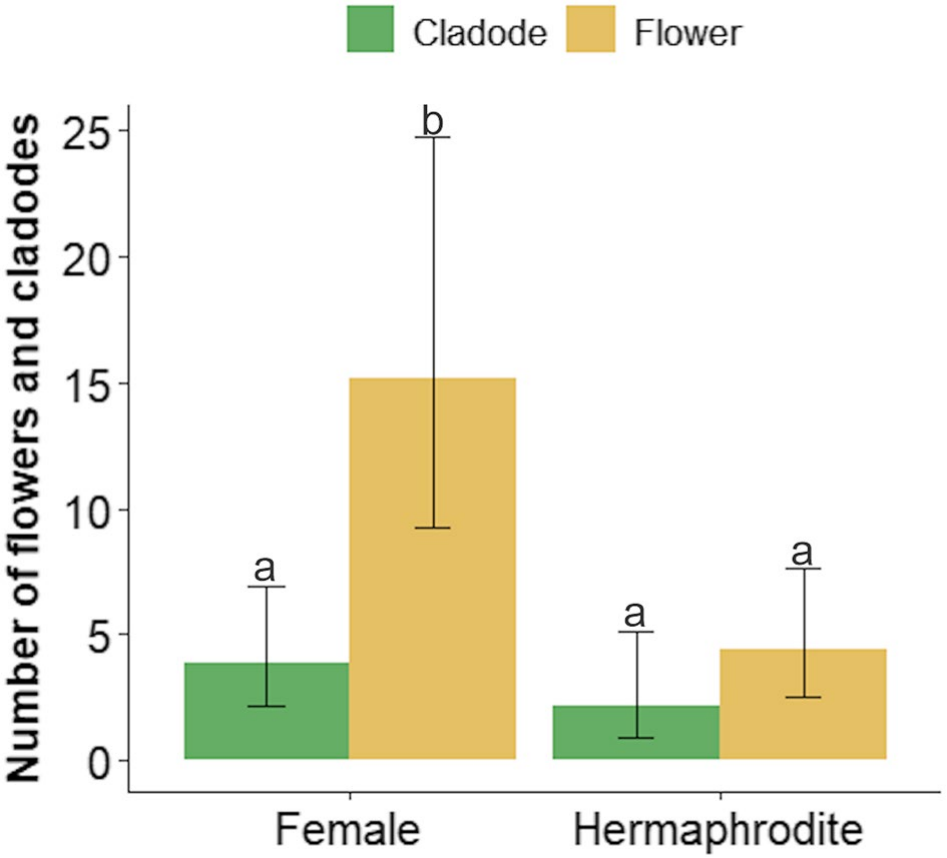
Comparison of the number of flower and cladode buds between sexual forms of *Opuntia robusta* in central Mexico. We fitted a generalized linear model with negative binomial error distribution. Different letters denote significant differences between the mean values at *P* ≤ 0.05.

**Figure 4:**
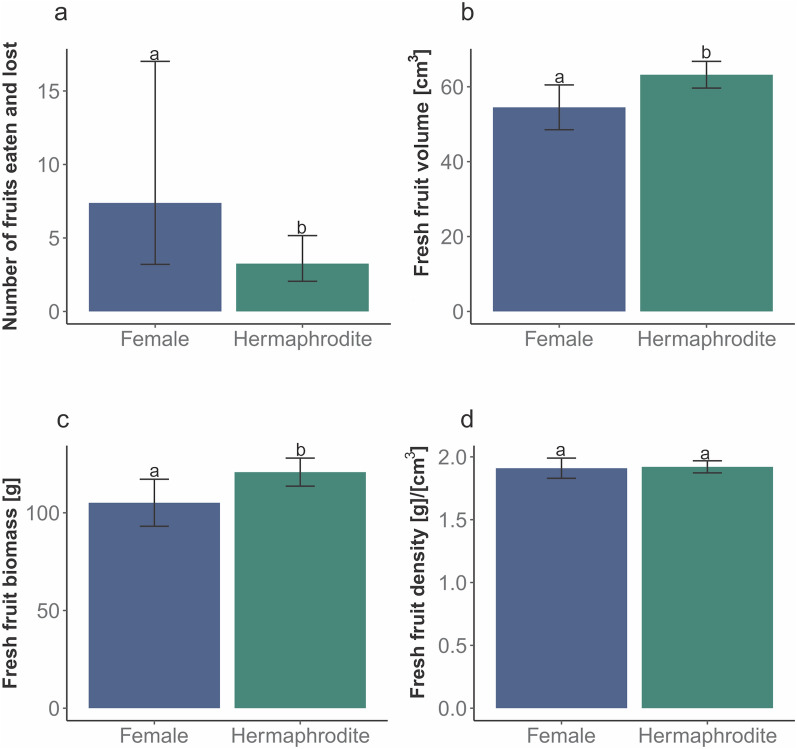
Comparison of the reproductive biomass traits for hermaphrodite and female *Opuntia robusta* plants growing in central Mexico. We estimated a) the number of fruits eaten by fructivores in hermaphrodite and female individuals, as well as b) the fruit volume [cm^3^], c) fruit biomass [g], and d) fruit tissue density [g × (cm^3^)^−1^]. More female fruits were consumed than hermaphrodite fruits (**a**). The average volume and average biomass of the ripe hermaphrodite fruits was significantly greater than that of the female fruits (**b** and **c**). Average fruit density was not significantly affected by sexual forms (**d**). We fitted a generalized linear model for fruit number with negative binomial error distribution, as well as for the volume, fresh biomass, and density, using the Gaussian distribution and a log link function. Different letters denote significant differences between the mean values at *P* ≤ 0.05.

Females presented with significantly more flowers per plant than hermaphrodites (Fig. 3; Table S3 online^32^). An analysis of data from the study by Janczur et al.^18^ showed that the number of female fruits eaten by fructivores was significantly higher at P ≤ 0.05 than that of hermaphrodite fruits (Fig. 4a). Fruits produced by hermaphrodite individuals had a significantly higher average volume and biomass than those produced by female plants (Fig. 4a, c). However, the average fruit density (Fig. 4d) and number of fruits collected (H = 1.198, N = 60, *P* = 0.3) did not differ significantly between sexual forms at *P* ≤ 0.05 (Table S4).

### Do female individuals have a higher occurrence/concentration of secondary metabolites than hermaphrodite individuals?

4-HBA, CGA, and QUE were present in approximately 40%, 20%, and 30% of female cladodes, and in 15%, 13%, and 7% of hermaphrodite cladodes, respectively. The highest concentrations of 4-HBA, CGA, and QUE were 8.34, 31.48, and 14.98 mg/mL of solution, respectively (Table S2; Table S1 online^31^).

The presence of 4-HBA and QUE was significantly affected by the sexual form, while that of CGA was not significantly affected at *P* ≤ 0.05 (Table 1a; Table S3 online^31^). The concentrations of 4-HBA were lower in females than in hermaphrodite individuals, while those of QUE were higher in females than in hermaphrodite individuals (Table 1b; Table S5 and Figure S5).

**Table 1:** Effects of sexual form (female, hermaphrodite), sampling month, cladode age class, cladode size (length, width, and thickness), number of cladodes above a given cladode, and order of cladodes above the soil, on the detection probability (**a**) and concentration (**b**) of 4-hydroxybenzoic acid (4-HBA), chlorogenic acid (CGA), and quercetin (QUE) for *Opuntia robusta* in central Mexico.

Type III tests of fixed effects														
(a)														
	4-HBA						CGA						QUE	
Effect	Df		F		*P*		F		*P*		F		*P*	
Sex	1		5.31		**0.02**		1.13		0.29		3.78		**0.05**	
Month	7		2.45		**0.02**		1.01		0.43		3.02		**0.004**	
Age class	1		4.63		**0.03**		1		0.32		**0.05**		0.8	
Size	3		2.08		0.1		0.20		0.90		2.60		**0.05**	
Cladodes above	1		9.96		**0.002**		6.57		**0.01**		**0.01**		0.9	
Cladode order from soil	1		0.4		0.53		0.16		0.69		0.34		0.6	
Significance test for parameter estimates in the logistic regression for cladode traits														
	Df		t		*P*		t		*P*		t		*P*	
Cladode length	1		− 2.23		**0.03**		− 0.25		0.8		− 2.6		**0.01**	
Cladode width	1		1.33		0.18		0.16		0.9		1.77		0.08	
Cladode thickness	1		0.84		0.4		− 0.74		0.5		0.84		0.4	
(b)														
		4-HBA						CGA						QUE
Effect		Df		F		*P*		F		*P*		F		*P*
Cladode order from soil		1		0.16		0.69		1.76		0.19		2.66		0.11
Sex		1		3.32		0.07		1.16		0.29		5.16		**0.03**
COS × Sex		1		7.02		**0.01**		1.66		0.20		1.81		0.19
Cladodes above		1		0.19		0.67		0.12		0.73		0.79		0.38
Sex		1		6.25		**0.01**		0.13		0.72		1.07		0.31
Cladodes above × Sex		1		3.8		**0.05**		0.03		0.87		0.12		0.73
Month		7		1.36		0.24		1.28		0.29		0.61		0.44
Sex		1		3.93		**0.05**		0.10		0.75		7.47		**0.01**
Month × Sex		7		1.08		0.39		0.50		0.83		2.69		0.11
Age class		1		0.01		0.94		0.55		0.46		0.15		0.70
Sex		1		6.99		**0.01**		0.37		0.55		1.08		0.30
Age class × Sex		1		5.29		**0.02**		0.77		0.38		0.08		0.77
Significance test for parameter estimates for cladode traits														
		Df		t		*P*		t		*P*		t		*P*
Cladode length		1		0.28		0.60		0.12		0.74		2.69		0.11
Cladode width		1		0.70		0.41		0.03		0.86		5.31		**0.03**
Cladode thickness		1		0.02		0.89		0.72		0.40		0.29		0.59

The three estimators of cladode size (length, width, and thickness) were considered jointly in the type III test of the fixed effect for the probability of detection. Then, their effects were examined separately, and significance tests were performed, in the logistic regression model; df—the number of degrees of freedom in the model. We fitted four different models for the concentrations of each metabolite, for cladode order from the soil (COS), cladodes above a given cladode, month, and age. The values of *P* for effects significant at *P* ≤ 0.05 are marked with bold text.

The occurrences of 4-HBA and QUE observed monthly were significantly affected by the sexual form. Neither the occurrence of CGA nor the concentrations of the three secondary metabolites were changed significantly on a monthly basis at *P* ≤ 0.05 (Table 1; Results S2 and Figure S3).

### Are female individuals less damaged than hermaphrodite individuals?

**Figure 5:**
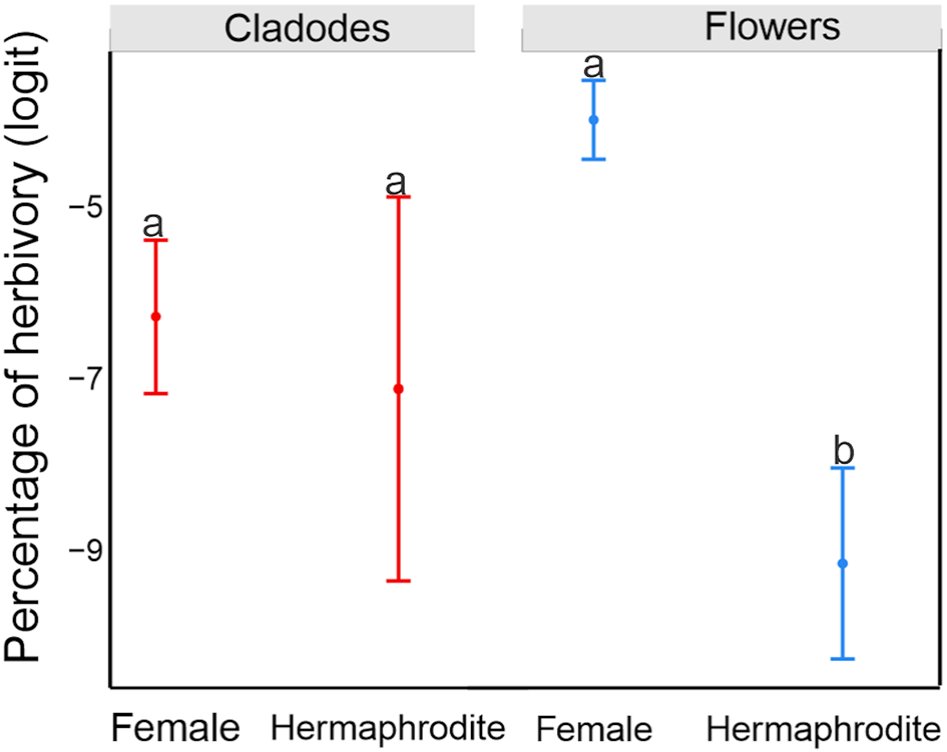
Effects of sexual form and plant structures on cumulative herbivory [proportion]. The figure illustrates the estimated mean values and confidence intervals obtained from a GLM, with Gaussian error distribution and identity link function. Cumulative herbivory was logit-transformed. The explicative variables were sex, type of structure, and their interactions. The difference between the female and hermaphrodite flowers was significant (*P* ≤ 0.001). Different letters denote significant differences between the mean values at *P* ≤ 0.05.

The herbivory rates of the cladodes were not significantly affected by sexual form. However, the herbivory rate for female flowers was significantly higher than that for hermaphrodite flowers at *P* ≤ 0.05  (Fig. 5, Table S6; see Dataset and Tables of the GLM results in Sandoval and Janczur^29^).

### Do younger vegetative tissues have a higher occurrence/concentration of secondary metabolites than older vegetative tissues?

**Figure 6:**
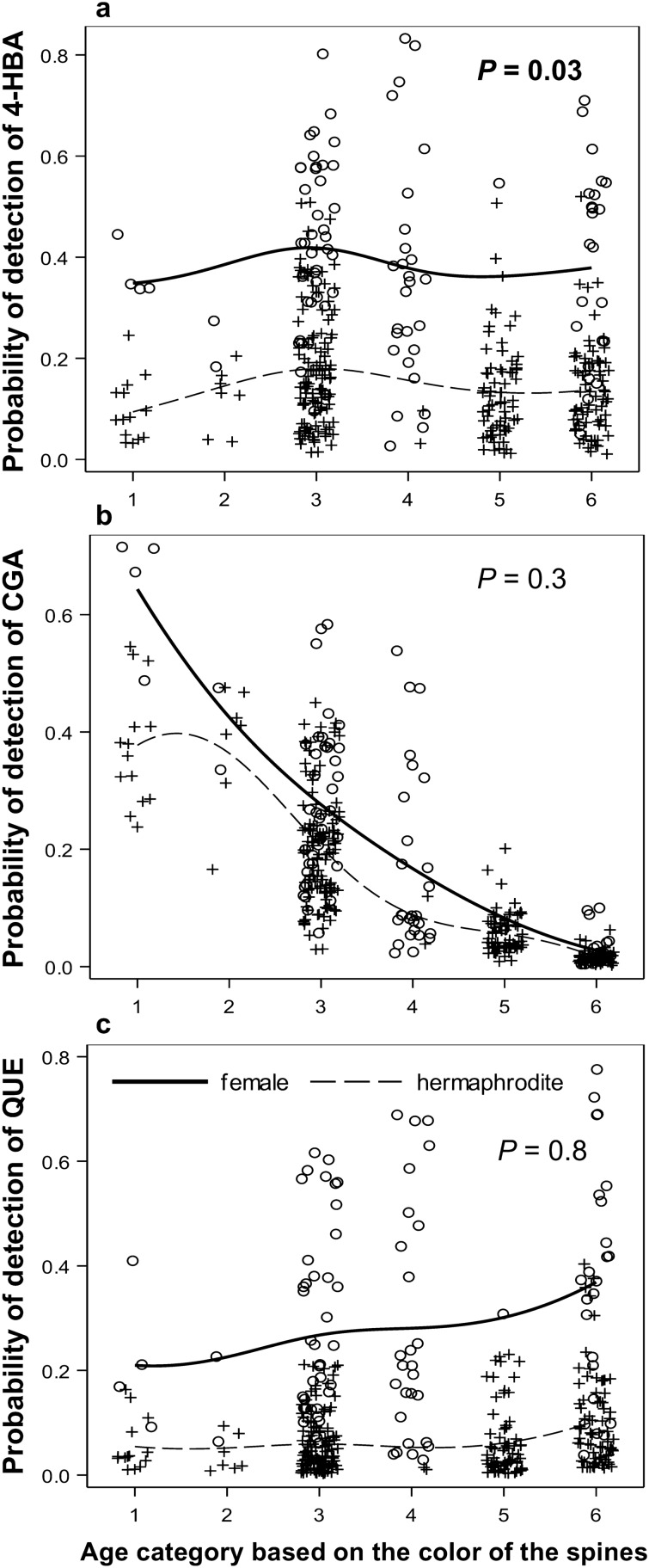
Effects of cladode age class on the proportion of cladodes containing **a—**4-hydroxybenzoic acid (4-HBA), **b—**chlorogenic acid (CGA), and **c—**quercetin (QUE) in the vegetative tissues of female and hermaphrodite cladodes. The detection probability of 4-HBA showed the pattern previously predicted by Janczur’s (2009) model. *P—*probability of adjustment in the logistic regression model for pooled sexual forms. Cladode age classes are based on the spine colour: 1*—*yellowish, 2*—*yellow, white base, 3*—*white yellowish, 4*—*white, 5*—*greyish, 6*—*black, with ‘1’ and ‘6’, being the youngest and oldest age classes, respectively. Significant relationships at *P* ≤ 0.5 are marked with bold text.

The cladode order from the soil (*x*_1_) and the number of cladodes above a given cladode (*x*_2_) were reliable estimators for the cladode age class (*y*) (*y* = -0.71*x*_1_ + 5.40, R^2^ = 0.62, and *y* = 0.72*x*_2_ – 1.22, R^2^ = 0.62, respectively, *P* < 0.0001 for both). However, almost 40% of the variance could not be explained in both the relationships. As there were no intersexual differences in either relationship, we pooled the data for both sexes (Figure S1; Tables S7, S8, and S9 online^31^). The proportion of cladodes containing 4-HBA was significantly lower in young cladodes than that observed in middle-aged and old cladodes (Fig. 6a). The proportion of cladodes containing either CGA or QUE was not significantly affected by cladode age (Fig. 6b and c). The proportion of cladodes containing either 4-HBA or QUE was significantly higher in female cladode group than that in hermaphrodite cladode group at *P* ≤ 0.05 (Fig. 6a and c; Table 1a; Tables S3, and S4 online^31^).

**Figure 7:**
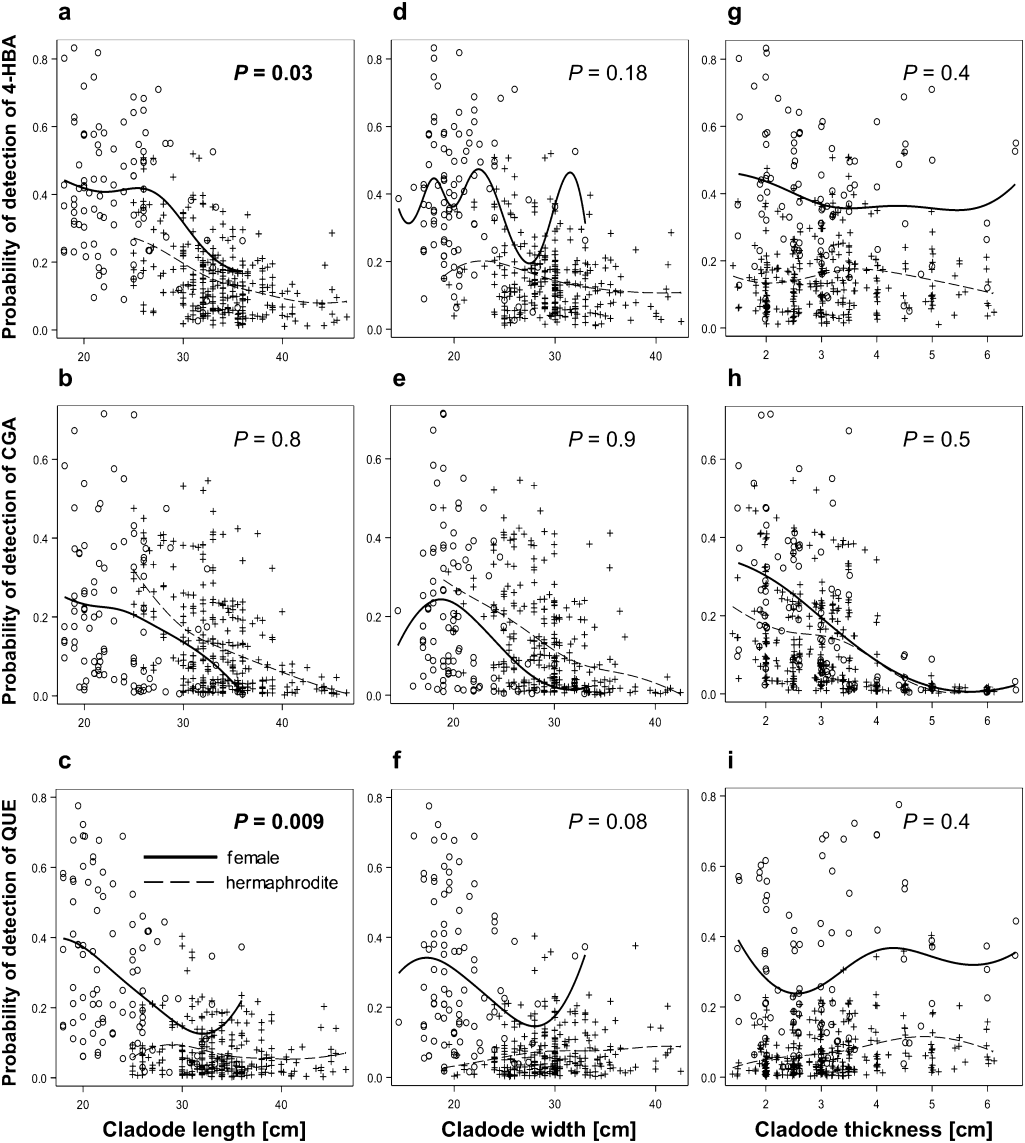
Effects of cladode length (**a**, **b**, **c**), width (**d**, **e**, **f**), and thickness (**g**, **h**, **i**) on the detection probability of 4-hydroxybenzoic acid (4-HBA), chlorogenic acid (CGA), and quercetin (QUE) in the vegetative tissues of female and hermaphrodite cladodes. 4-HBA and QUE had higher detection probabilities in the shorter cladodes. Female cladodes had a higher detection probability for 4-HBA and QUE than hermaphrodite cladodes of the same length. There was no intersexual difference in the detectability of CGA. *P—*probability of the adjustment in the logistic regression model for pooled sexual forms. Significant relationships at P ≤ 0.5 are marked with bold text.

Shorter cladodes exhibited higher detection probabilities for 4-HBA or QUE than longer cladodes (Fig. 7a and c). The proportion of cladodes containing CGA was not significantly affected by cladode length (Fig. 7b). The probability of detecting these three secondary metabolites in the cladodes was not significantly affected by cladode width or thickness at *P* ≤ 0.05  (Fig. 7d-i). Female cladodes had a higher detection probability for the three secondary metabolites than hermaphrodite cladodes with similar cladode lengths (Fig. 7a and c; Table 1a and S2; Tables S4, S3, and S4 online^31^).

**Figure 8:**
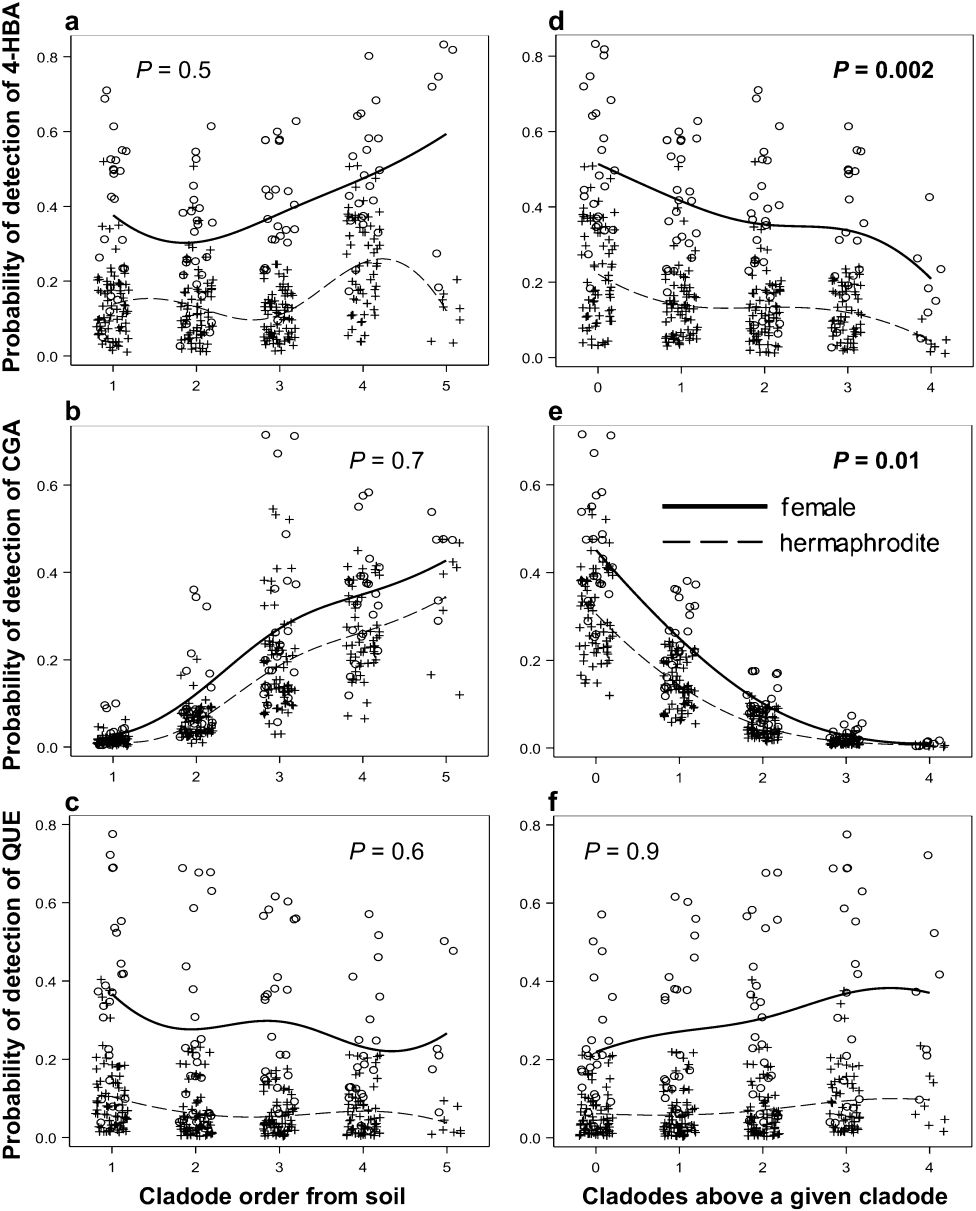
Effects of cladode order when evaluated from the soil level or effects of the number of cladodes above a given cladode on the proportion of cladodes containing **a**, **b—**4-hydroxybenzoic acid (4-HBA), **b**, **e—**chlorogenic acid (CGA), and **f**, **c—**quercetin (QUE) in the vegetative tissues of the female and hermaphrodite cladodes. The detection probability of 4-HBA and QUE was lower in cladodes with more daughters. *P—*probability of adjustment in the logistic regression model for pooled sexual forms. Significant relationships at P ≤ 0.5 are marked with bold text.

The proportion of cladodes containing secondary metabolites was not significantly affected by cladode order (Fig. 8a–c). Parental cladodes with fewer daughters had a higher detection probability for 4-HBA or CGA than those with a higher number of daughters (Fig. 8d and e). This relationship was not significant for QUE (Fig. 8f; Table 1). Overall, female cladodes had a higher detection probability for 4-HBA and QUE than hermaphrodite cladodes; however, this difference was not significant for CGA at *P* ≤ 0.05 (Fig. 8a–f; Tables 1a and S2; Tables S1, S3, and S4 online^31^).

Cladode age had no effect on the concentrations of these three secondary metabolites. In case of hermaphrodites, 4-HBA concentrations were significantly higher in younger cladodes than in older ones. Hermaphrodite cladodes had higher 4-HBA concentrations than those observed in female cladodes (Table 1b; Figure S4). There was a significantly negative relationship between hermaphrodite cladode length and width, and 4-HBA concentrations. CGA concentrations were higher in wider hermaphrodite cladodes. QUE concentrations were significantly higher in female cladodes than in hermaphrodite cladodes and were lower in wider cladodes. Cladode age did not significantly affect the other parameters at *P* ≤ 0.05 (Table 1b; Figures S5 and S6; Table S5 online^33^).

### Is there evidence of a trade-off between investments in defence, growth, and reproduction?

The relationships between sexual form and cladode length, cladode order, and cladode age maintained the same slope for both sexes (*W*_T_ = 2.385, *P* = 0.1; *W*_T_ = 0.017, *P* = 0.9). However, both relationships differed significantly in the intercept (*W*_T_ = 446.1, *P* < 0.001 and *W*_T_ = 449.2, *P* < 0.001, for the former and the latter relationship, respectively), as hermaphrodite cladodes of all orders and age classes were larger than the respective female cladodes (Figure S2; Tables S7 and S8 online^31^). Females had significantly more flowers per plant than those observed in hermaphrodites at *P* ≤ 0.05  (Fig. 3; Table S3; Dataset and Tables of the GLM results in Sandoval-Molina and Janczur^32^ online), and their flowers showed slower growth (Fig. 1). Additionally, female cladodes presented a higher occurrence of 4-HBA and QUE (Table 1a).

### Does the existence of trade-offs between different secondary metabolites affect the predictions of the plant defence hypothesis?

**Figure 9:**
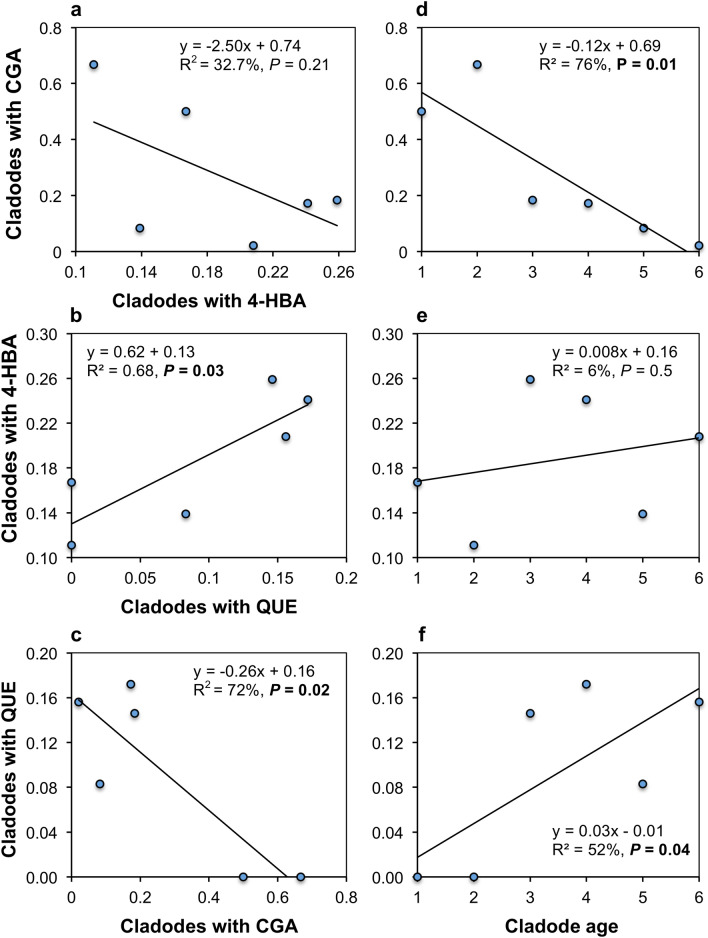
Relationship between the proportion of cladodes in the six age classes containing chlorogenic acid (CGA) and 4-hydroxybenzoic acid (4-HBA) (**a**), quercetin (QUE) and 4-HBA (**b**), and QUE and CGA (**c**), and the relationship between the average proportion of cladodes classified by age class, harbouring CGA (**d**), 4-HBA (**e**), and QUE (**f**). R^2^*—*coefficient of determination, *P—t* test probability for the coefficient of correlation. Significant relationships at *P* ≤ 0.5 are marked with bold text.

The age-dependent average proportion of cladodes containing CGA was negatively associated with that of cladodes containing 4-HBA; however, this relationship was not significant (Fig. 9a). The age-dependent average proportion of cladodes containing QUE was significantly and positively correlated with that of cladodes containing 4-HBA (Fig. 9b), but was significantly and negatively correlated with that of cladodes containing CGA (Fig. 9c). When cladodes were classified by age class, the proportion of cladodes containing CGA was found to be negatively correlated with cladode age (Fig. 9d). The same relationship regarding 4-HBA was positive but non-significant (Fig. 9e), and for QUE, the relationship was both positive and significant at *P* ≤ 0.05  (Fig. 9f; Table S6 online^31^).

## Discussion

### Are tissues of female individuals costlier than those of hermaphrodites?

This study has shown that the growth rate of the reproductive biomass of hermaphrodite individuals is higher than that of female individuals, indicating that hermaphrodites have a lower per biomass unit cost than females. Based on the findings reported by Lambers and Poorter^34^, the higher growth rate of hermaphrodites could have induced a higher intake of nitrate and phosphate to meet their demands, suggesting that the hermaphrodites are naturally fast-growing, compared to females. Additionally, hermaphrodite cladodes were significantly larger than female cladodes. Although the average per tissue biomass-unit-cost of the species was not significantly different, adult hermaphrodite cladodes were larger. This may be attributable to the fact that hermaphrodites allocate relatively fewer resources for reproduction, as compared to females.

An interspecific comparison showed that lower tissue carbon concentrations and dry matter percentages were associated with a higher relative growth rate, with slight differences in carbon concentrations resulting in considerable differences in the relative growth rate^34,35^. Furthermore, glucose requirements decrease with an increased relative growth rate^34^. These results are in accordance with those obtained in previous studies comparing the relationship between the growth rate and phosphate uptake rate of fast- and slow-growing plants, which have shown that under optimal nutrient supply conditions, fast-growing species exhibit the highest nutrient uptake rate per unit root weight^36,37^.

### Do female individuals have a higher secondary metabolite occurrence/concentration than hermaphrodite individuals?

Meteorological variables did not significantly affect the occurrence and concentrations of the three secondary metabolites in hermaphrodite plants, but significantly affected 4-HBA and QUE concentrations in female plants. We hypothesised that this could be attributed to the higher energy demands associated with tissue production in females, as compared to that by hermaphrodites; this was supported by the lower relative growth rates of female plants.

Additionally, the higher energy costs of females may be responsible for the lack of association between their secondary metabolite concentrations, and this may also explain the negative non-significant associations established between concentrations of QUE and 4-HBA or CGA. The fact that hermaphrodites can simultaneously increase the concentrations of the three metabolites reinforces this hypothesis, as hermaphrodite individuals spend relatively lower amounts of energy in the development of vegetative tissues, compared to females, and can invest more energy in the production of these three secondary metabolites without compromising the production of other compounds. In female cladodes, the occurrence and concentrations of QUE were positively associated with global radiation, and negatively associated with potential evapotranspiration; both secondary metabolite traits of 4-HBA were negatively and positively associated with global radiation and potential evapotranspiration, respectively, suggesting the existence of a trade-off of resources for the production of 4-HBA and QUE. The negative response of 4-HBA in the female cladodes to global radiation, and the lack of such responses in hermaphrodite cladodes resulted in higher 4-HBA concentrations in hermaphrodites than those reported in females.

The effects of global radiation and potential evapotranspiration on the proportion of cladodes containing secondary metabolites were different between the sexual forms. Global radiation and potential evapotranspiration exerted an inverse effect on 4-HBA and QUE concentrations in female cladodes. The occurrence of 4-HBA and QUE was significantly higher in female cladodes than in hermaphrodite cladodes. These differences in metabolite occurrences in the different sexual forms could be attributed to differences in their energy allocation. However, the occurrence and concentrations of CGA in the two sexual forms cannot be attributed to the effects of the meteorological variables or energy allocation because in both sexual forms, this metabolite did not respond to changes in these abiotic variables.

The detection probability of 4-HBA and QUE was higher in female cladodes of all orders, compared to hermaphrodite cladodes, which was consistent with the predictions of the optimal defence hypothesis. Sexual form and cladode length did not affect the above-mentioned findings, as the detection probability of 4-HBA and QUE was also higher in female cladodes, compared to hermaphrodite cladodes of the same length.

If females and hermaphrodites differ in their costs for producing vegetative and/or reproductive tissues, one of the existing hypotheses (the optimal defence hypothesis or the optimal defence hypothesis + the resource availability hypothesis) may be tested by comparing the sexual forms. In this study, although hermaphrodite cladodes were larger than female cladodes, female cladodes had higher 4-HBA and QUE detection probabilities and concentrations. The larger cladode sizes of the hermaphrodite plants could be attributed to their relatively lower levels of resource allocation for reproduction. Furthermore, hermaphrodite fruits were significantly larger than female fruits; however, fruit density was not significantly affected by sexual form^18^. In this study, we observed that there was a relatively higher resource allocation for reproduction and vegetative growth relative to defence in hermaphrodite plants, and this was evidenced by the faster growth rate of hermaphrodite fruits and the similar fruit tissue densities of female plants. The detection probability of 4-HBA and QUE was higher in the slower-growing sexual forms than that observed in the faster-growing forms, which supported the predictions of the resource availability hypothesis. This also supported the optimal defence hypothesis which states that the slower-growing sexual form may have a higher energy cost. The higher tissue costs of the female fruits imply a higher nutritional value for fructivores. This offers a plausible explanation for the increased consumption of ripe female fruits by fructivores, compared to the consumption by hermaphrodite fruit eaters; this phenomenon increases the dispersal probability of female fruit seeds.

The concentration of 4-HBA was lower in female cladodes than that in hermaphrodite cladodes, which was consistent with the resource competition hypothesis. Additionally, female tissues presented with slower growth rates and higher levels of herbivore damage than hermaphrodite tissues. However, this finding was contrary to the optimal defence hypothesis, as the occurrence of 4-HBA was found in higher proportions in the female cladodes. However, the concentration and occurrence of CGA was not significantly affected by sexual form, which was not in agreement with the resource availability hypothesis or the optimal defence hypothesis.

When comparing both sexual forms, the inverse relationship between 4-HBA concentrations and the proportion of cladodes harbouring it might be attributable to the existence of a trade-off or different defensive strategies displayed by the sexual forms. Hermaphrodite plants were not defended in a manner akin to female plants, as the occurrence of 4-HBA was limited to a few cladodes; however, in a few cladodes, its concentrations were high. To the best of our knowledge, the energy production cost of 4-HBA in vivo is unknown. Therefore, we could not conclude if this inverse relationship for both sexual forms regarding its concentrations and probability of detection was an expression of a trade-off or a ‘decision’ of the plant on the manner of distributing 4-HBA (i.e. higher concentrations when there are few cladodes and lower concentrations when there are more cladodes). The latter could be possible if the occurrence of the secondary metabolite was dynamic during the eight-month study period. Secondly, we observed in a previous study that 4-HBA could be transferred from apical to basal cladodes^38^.

QUE concentrations and distribution in the cladodes exhibited similar patterns. As QUE moves from apical to basal cladodes^38^, a trade-off between the concentration and the proportion of cladodes harbouring it may have occurred; however, this was not observed in this study. The distribution pattern of QUE is species-specific. Furthermore, the occurrence of QUE in the plant was dynamic during the eight-month study period.

Only a few studies showed intersexual differences in secondary metabolites content, however, they regard dioecious plants (female and male individuals). For example, the only study showing higher concentration of 4-HBA, CGA and QUE in female than in male plants was the study of Dziedzic et al.^14^ carried out on *Rumex thyrsiflorus*. The study of Iszkuło et al.^15^ showed higher concentration of taxane diterpenes in female than in male individuals of *Taxus baccata*. Both results are consistent with the predictions of the optimal defence hypothesis. On the other hand, Massei et al.^16^ found that males of *Juniperus oxycedrus* grew faster than females but also had higher concentrations of both phenolics and terpenoids, suggesting that allocation of resources to reproduction in plants reduced the resources available to both growth and secondary compounds, a result consistent with the predictions of the resource competition hypothesis. These contradictory results point to the need to carry out future studies that can fill the gap in the knowledge of the defence pattern in plants with different sexual forms.

### Are female individuals less damaged than hermaphrodite individuals?

The average proportion of damaged surface area was higher for female flowers than that observed with hermaphrodite flowers. The results of the intersexual comparisons of relative growth rate and cumulative herbivory were not consistent with the predictions of the optimal defence hypothesis, as a more energetically expensive sexual form exhibited a higher extent of damage. Our results were also not in line with the predictions of the resource availability hypothesis, as faster-growing cladodes were less damaged than slower-growing ones. Additionally, our results were not compatible with the predictions of the resource competition hypothesis. This was because even if female individuals invested more resources into reproduction and grew at a slower rate, they exhibited a higher occurrence of two out of three secondary metabolites than hermaphrodite individuals and the same occurrence of one metabolite as hermaphrodite individuals. However, as per the predictions of this hypothesis, a more costly sexual form exhibited a higher extent of damage. These results were not attributable to the competition between the costs of reproduction/growth and production of secondary metabolites, as the costlier sexual form invested more resources in secondary metabolite synthesis.

### Do younger vegetative tissues have a higher occurrence/concentration of secondary metabolites than older vegetative tissues?

The proportion of cladodes harbouring 4-HBA and CGA showed a similar pattern as that predicted using the Janczur’s model^3^ for both sexual forms, i.e. the proportion of cladodes harbouring 4-HBA and CGA was higher in shorter and narrower cladodes, in those with younger spines, and in those from either a higher order or those with fewer daughter cladodes. In certain cases, as predicted using the Janczur’s model^3^, the aforementioned proportion was lower for the age estimator associated with a younger age class, and in certain cases, such a phenomenon was not observed. This may have occurred because Janczur’s model presents solutions for cases where the concentration of defence chemicals is null at the beginning of plant (organ) life. In this study, we estimated secondary metabolite levels in grown cladodes, which already contained secondary metabolites. CGA showed a more similar pattern to that predicted by the optimal defence hypothesis for the age of the vegetative tissues; CGA was found more frequently in younger cladodes than in older ones. Almost no cladodes with black spines, which were demonstrably the oldest, contained CGA. Adoption of the Janczur’s model helped examine the effects of the proportion of tissue loss and not of other types of damage on the plant’s defence pattern; however, we hypothesised that both these metabolites were produced for defensive purposes. The proportion of cladodes harbouring QUE showed different patterns for different cladode age estimators; cladode length demonstrated a similar tendency (higher occurrence in shorter cladodes), but cladode age based on spine colour and cladode position on the branch showed an inverse non-significant tendency.

In hermaphroditic cladodes, the relationship between cladode age and secondary metabolite concentration was significant only for CGA. Overall, the concentration of CGA was inversely related to the occurrence of CGA in the cladodes, suggesting a trade-off between investment in cladode formation and CGA biosynthesis. The existence of this trade-off was even more probable because neither the proportion of cladodes with CGA nor its concentrations were affected by meteorological factors. These findings are contrary to the predictions of the optimal defence hypothesis. The lack of difference in the occurrence of QUE between the cladodes of different orders and those with different numbers of daughters, as well as the existence of a strong positive relationship between the age-classified occurrence of QUE and cladode age, demonstrate a transfer from the more photosynthetically active cladodes to the less photosynthetically active (older) ones. This scenario is more probable compared to the supposed equally intensive photosynthesis in cladodes from different orders.

Even when larger cladodes were generally older than smaller ones, approximately 30% of the relationship between cladode age and cladode order or number above a given cladode was explained by other factors. These phenomena resulted in a lack of congruence in predictions based on different cladode age estimators. Plant should perform adjustments to produce defensive substances in terms of their relative value in terms of fitness, rather than in terms of age or size. A measure of the relative value for both vegetative and reproductive tissues should be considered; however, first, the criteria for such a relative measure should be established.

### Is there evidence of a trade-off between investment in defence, growth, and reproduction?

Higher relative resource allocation for reproduction (higher relative reproductive investment) and secondary metabolite biosynthesis in female individuals was associated with lower resource allocation for growth. The probability of female flowers growing on cladodes protected by secondary metabolites was higher than that for hermaphrodite flowers, because female flowers were conferred with more protection by secondary metabolites than hermaphrodite flowers. This is consistent with the predictions of both the optimal defence hypothesis and resource availability hypothesis, as more valuable female structures will be conferred with more protection by secondary metabolites. Moreover, these structures were exposed to more damage, and this finding was consistent with the predictions of the resource competition hypothesis. Increased levels of damage to the female reproductive structures, irrespective of their higher cost, would be disadvantageous if reproductive success is not considered. For this strategy to be optimal, higher relative reproductive investment in female plants and a higher zoochory rate in this sexual form should be positively correlated to fitness and offset tissue loss.

### Does the existence of trade-offs between different secondary metabolites affect the predictions of the plant defence hypothesis?

Based on statistical analysis of the age-classified cladodes, it can be implied that if the relationship between age estimators and the occurrence of secondary metabolites in cladodes not classified by age is weak or non-significant, and is either significant or inverse in age-classified cladodes, then classification by age exerts an effect on the distribution of the metabolites among cladodes. For example, as the occurrence of 4-HBA was inversely associated with age when compared across all cladodes and was slightly and positively associated with age when compared across age-classified cladodes, we concluded that older cladodes received this metabolite from the higher-order cladodes; however, this flow was variable and small on average. Analogously, as the relationship between cladode age and QUE occurrence was markedly flat and non-significant and presented with a highly positive and statistically significant slope for age-classified cladodes, we could conclude that there was a significant flow from higher-order to lower-order cladodes. Furthermore, as the occurrence of CGA showed a similar relationship with age for both age-classified and non-age-classified comparisons, we could conclude that age classification did not influence CGA distribution among the cladodes of the different age classes. In a previous study, we showed that QUE was produced in apical cladodes and moved to basal cladodes in a statistically significant manner, and 4-HBA moved in the same direction in a statistically non-significant manner. Additionally, CGA was produced autonomously by each cladode^38^. The transfer of QUE and 4-HBA from higher-order to lower-order cladodes is not concordant with the predictions of the existing defence hypotheses, since generally, younger plant organs are considered to be more valuable because of their higher photosynthetic potential. However, it is also possible that lower-level cladodes are more valuable in terms of fitness because their loss implies the loss of all daughter cladodes.

The negative relationship between the average age-classified proportion of cladodes harbouring CGA and QUE could be attributable to the fact that shikimic acid is a direct precursor of QUE, and a precursor of phenylalanine and cinnamic acid, with the latter also being direct precursors of QUE^10^. This implies that the synthesis of both phenylalanine and cinnamic acid should also be negatively correlated to the synthesis of CGA^39^. However, another probable explanation is that a stronger negative relationship between cladode age class and the average CGA occurrence in age-classified cladodes could explain 76% of the model, together with a weaker positive relationship between QUE age-classified occurrence. Then, 52% of cladode age could be explained with the adoption of the model and could create a negative relationship between both occurrences (the negative effect of CGA occurrence with cladode age was stronger than the positive effect of QUE occurrence). An analogous explanation can be offered for the non-significant negative relationship between age-classified occurrences of CGA and 4-HBA. SA was absent in all samples. Since 4-HBA is a precursor of SA, it is unclear why it was not converted to SA^40^. Another pathway may also be involved in the conversion of phenylalanine and trans-cinnamic acid to SA via benzoic acid, along with a parallel pathway involving phenylalanine, trans-cinnamic acid, 4-coumaric acid, and 4-HBA. Based on the involvement of this pathway, there may be competition over the common precursor (*trans*-cinnamic acid) during the processes of 4-HBA and SA synthesis. SA is known mainly as a signalling molecule involved in the induction of pathogenesis-related proteins^10^, and there are very few reports regarding the signalling function of 4-HBA^22,41^. we propose that the positive relationship between 4-HBA and QUE may be an outcome of the positive and significant relationship between the age-classified occurrence of 4-HBA or QUE and cladode age, owing to their relocation from younger to older cladodes. A positive and significant relationship for QUE and a less remarkable positive and non-significant relationship for 4-HBA resulted in a positive relationship between the age-classified occurrence of both metabolites. However, the signalling function of 4-HBA remains a possible explanation when considered together with the effect of metabolite relocation, since the relationship between the age-classified occurrence of both metabolites explained a greater variability (68%) than the relationships between the occurrence of each metabolite and cladode age (6% and 52% for 4-HBA and QUE, respectively). To the best of our knowledge, the signalling function of 4-HBA was observed only in cucumbers infected with *Pseudomonas syringae*^22^. Generally, 4-HBA is considered biologically inactive^41^. Differences observed in the relationships between the secondary metabolite pairs discussed in this section, and those discussed in the section concerning the effects of meteorological factors on secondary metabolite traits, were derived from the fact that, in the latter analysis, cladodes were not classified by age.

In another study, we showed that CGA exhibited low mobility between cladodes^38^. In hermaphrodite cladodes, our results showed an inverse relationship between the proportion of cladodes harbouring CGA and its concentration in tissues. These results are best explained as an expression of a trade-off during its synthesis. Decisions regarding the investment of lower concentrations of CGA in a higher number of cladodes, or higher concentrations of CGA in a lower number of cladodes may be undertaken accordingly. Independent of proximal causes, predictions based on both the concentration and proportion of cladodes were contradictory and concordant with different hypotheses.

## Conclusions

1. Consistent with both the optimal defence hypothesis and resource availability hypothesis, cladodes exhibiting slower growth (female) were more likely to contain 4-HBA or QUE than hermaphrodite cladodes.
2. Consistent with the resource competition hypothesis, the concentration of 4-HBA was higher in hermaphrodites.
3. The sexual form exhibiting faster growth presented with less damage, which was consistent with resource competition hypothesis.
4. Positive or negative relationships between secondary metabolite pairs may result from relocations between plant organs. The direction of this relocation may or may not be consistent with the predictions of the optimal defence hypothesis.
5. There is no evidence suggesting that the descriptive phrase, ‘higher levels of defence,’ as used in the hypotheses, is an accurate description of reality. The descriptive phrase used in these hypotheses is not adequately precise to refer to the extent of plant defence or to describe the concentrations of secondary metabolites in plant tissues.
6. The results of our study highlight the need to redefine hypotheses that attempt to explain the mechanisms by which the selective pressures of both biotic and abiotic factors shape the extent of chemical defence and damage by herbivores. Particularly, secondary metabolite levels in plant tissues and damage by herbivores may not be negatively correlated with each other, indicating that these traits are good predictors of the reproductive success of plants. However, for field studies, the only estimator that should be considered based on the new hypothesis is the lifetime reproductive success or estimators directly showing correlation with it.

## Methods

### Study area

We performed this study in San Nicolas Tecoaco village (20° 2′ 38.2ʺ N, 98° 35′ 16ʺ W), Hidalgo State, central Mexico, from March 2014 to October 2014. This location has an annual average temperature of 16 °C and an average altitude of 2600 m above sea level. The type of vegetation occurring in this area is classified as a xerophilous shrubland^42^.

### Study species

*Opuntia robusta* (Cactaceae) is an endemic plant found in Meridional Altiplano, México^43^, which exists in the following three sexual forms: hermaphrodite, dioecious (male and female), and trioecious^44^. In a parallel study, Sandoval-Molina^45^ found that the most common herbivores of this plant were leaf-footed bugs, *Chelinidea* sp., *Narnia* sp. (Hemiptera: Coreidae), the cactus long-horned beetle, *Moneilema* sp. (Coleoptera: Cerambycidae), and mining insects. Before 2017, this population was considered to be gynodioecious; thus, we did not collect samples from male individuals in this study. In 2018, fewer than 15 male individuals were reportedly present in a population of more than 800, and most of these were hermaphrodites (Supplementary Information).

### Determination of plant sex

White empty anthers, short style, and well-developed lobular stigma characterised female flowers, while a relatively longer style compared to that of the female and functional anthers characterised hermaphrodite individuals^44^.

### Comparison of tissue cost between female and hermaphrodite individuals

In March 2017, we undertook a census in San Nicolas Tecoaco, to identify the number of female and hermaphrodite plants with cladode and flower sprouts from the set of plants studied in the previous years. We selected 1–2 m tall plants, located 5–10 m apart for sampling. Finally, we randomly selected 19 plants (eleven female and eight hermaphrodite individuals) bearing flower buds and young cladodes on different branches for analysis and tagged the cladodes and flower sprouts using a permanent marker. We marked the flower sprouts on the adjacent side of their parental cladode surface.

Between March 2017 and June 2017, we obtained sufficient data to estimate the relative growth rates of the species, in order to explore possible differences in the energy costs of cladodes and flower buds between the two sexual forms of *O. robusta*. We measured the length, width, and thickness of each cladode and flower bud twice during the study, once at the beginning, and once at the end of the study. Additionally, we also measured the lengths of the flowers from the base to the beginning of the sepals. Since the flower buds were spherical, we considered the thickness to be equal to the width. Subsequently, we calculated the flower volume immediately after the emergence of cladodes and flower buds, and the final volume after anthesis. We estimated the initial and final volumes (*V*_*x*_) of the cladodes using the formula *V*_x_ = ((*a*/2))/((*b*/2)*π*)*c*, and those of the flowers using the formula *V*_x_ = 4/3π*a*^2^*b*. Here, *x* represents the time of measurement (initial or final), *a* and *b* represent the major and minor axes of the ellipsis, while *c* represents the cladode thickness. We measured all estimators to the nearest 1.0 mm and represented values in centimetres. We estimated the relative growth rate (RGR) using the formula proposed by Hunt^46^: RGR = (ln*V*_f_ – ln*V*_i_)/(*t*_2_ – *t*_1_). Here, *V*_f_ represents the final volume [cm^3^], *V*_i_ represents the initial volume [cm^3^], *t*_1_ represents the initial time [day], and t_2_ represents the final time [day].

We compared relative growth rate data using a generalized linear model (GLM) with gamma error distribution in the R software, using the log link function^47^. The explanatory variables included sex, type of structure, and their interactions. We performed partial regression using the *ggeffects* package in R^48^.

We obtained meteorological variables, including total precipitation [mm], maximum temperature [°C], minimum temperature [°C], mean temperature [°C], global radiation [W(m^2^)^−1^], relative humidity [%], reference evapotranspiration [mm], and potential evapotranspiration [mm] for the Singuilucan municipality from March–October 2014, from the official Mexican Government weather station database of the Instituto Nacional de Investigaciones Forestales Agrícolas y Pecuarias^49^. We summed up the data for the per-day total precipitation, and that for the reference and potential evapotranspiration, from the beginning of each month through the sampling day. In the months (March, April, and May) or days when values from the meteorological database were underestimated, we averaged the values for the closest preceding and following days. If we lacked the data for more than one day and the data for such days could not be acquired, we considered a repetition of the averaged value for the days for which we lacked data, between the existing days. For July, we considered the values for the previous day (11/07/14), since we lacked the data for the days on which sampling was performed and the subsequent days. For the additive variables (total precipitation, reference, and potential evapotranspiration), we summed up data for 30 days, excluding data for one day, for the 31-day period.

To determine the effects of the environmental variables on the concentration and presence/absence of secondary metabolites, we used R to formulate a structural equation model (SEM) in piecewiseSEM^47,50^. For concentrations, we fitted linear mixed-effects models using the *nlme* package^51^ and used the plant ID as a random factor. To evaluate the presence or absence of substances, we fitted generalized linear models with binomial error distributions and logits as the link functions. The concentration and presence/absence of 4-HBA, CGA, and QUE were dependent variables, and total precipitation, average temperature, global radiation, relative humidity, and potential evapotranspiration were explanatory variables. We analysed the sexes separately, and the substance concentration variables were log + 1 transformed. We assessed the goodness-of-fit using the Fisher function in the *piecewiseSEM* package^50^, where a larger *p*-value implies better data adjustment to the model. We conducted a visualisation of the SEM models using Biorender^52^, flaticon^53^, and CorelDRAW^54^.

We estimated fruit traits (biomass [g], volume [cm^3^], and tissue density [g × cm^−3^]) and the number of fruits eaten by fructivores and compared them between the sexual forms using data reported by Janczur et al.^18^. The former comparison enabled the assessment of the possible differences in reproduction per fruit biomass between the sexual forms. The latter comparison enabled the assessment of the differences in preference for fruits eaten by animals in relation to the different sexual forms, and thus, the mechanisms by which this may increase the probability of seed dispersal. Higher zoochory of one sexual form may occur not only because of differences in fruit biomass density [g × cm^−3^], but also because of differences in the volatile substance content between the sexual forms.

To test the effects of sexual form on fruit traits, we used generalized linear models in R. To analyse the number of fruits eaten, we used the negative binomial error distribution and log link function, and the Gaussian error distribution and identity link function for the other fruit traits^47,55,56^. We performed all post-hoc contrasts for fruit traits using the *emmeans* package^47,57^, and generated plots using the *ggplot* R package^47,58^. We compared the average number of fruits produced by the two sexual forms using the Kruskal–Wallis test.

### Comparison of secondary metabolite occurrence/concentration between female and hermaphrodite individuals

We obtained plant samples for secondary metabolite analysis using 100 m long Canfield lines, which were parallel to the contours of the hill and located 60 m from each other, and selected plants that were located near the lines and were 10 m apart for analysis. We randomly assigned each plant to one of the eight groups established herein, with three female plants and twelve hermaphrodite plants. The uneven number of individuals of each sex was attributable to the low proportion of females in the population. We tagged examined cladodes on their surface using a permanent marker.

We used a stainless-steel punch (Ø = 0.5 cm) to remove two samples of vegetative tissue from cladodes belonging to the same order of each plant. We perforated the mid-section of the arc delimited by the border of the upper quarters of the cladodes, approximately 1 cm away from the edge. We placed samples in labelled Ziploc bags, stored them in a cooler containing ice, and then transported them to the laboratory in a portable refrigerator at − 20 °C. The samples were stored in the laboratory at − 40 °C until extraction.

We performed homogenisation of approximately 1 g of the sample containing the cuticle in 35 mL of 100% methanol in an ultrasonic 6 L bath for 30 min at room temperature (21 °C). We filtered the methanol extracts, placed them in amber bottles, and stored the bottles at − 20 °C until further analysis^59^. We determined the types and concentrations of secondary metabolites in these tissues using high-performance liquid chromatography (HPLC), in accordance with the procedure described by Janczur and González Camarena^59^, using the following: Waters 717 liquid chromatograph with autosampler, Waters 2487 HPLC Absorbance UV–Vis Detector, Waters 1525 Binary HPLC Pump, Waters control module with SAT/IN Bus (Waters, Milford, MA, USA), Symmetry HPLC C18 column (particle size 5 µm, length 250 mm, internal Ø = 4.6 cm; Waters, Milford, MA, USA). We filtered the extracts using a 0.45 µm pore size nylon-membrane filter. The mobile phase consisted of 0.1% v/v acetic acid (A) together with 100% acetonitrile (B). For the mobile phase A, we dissolved 1 mL of glacial acetic acid with HPLC water, until the volume was 1 L. For the mobile phase B, we used 100% acetonitrile. We filtered both mobile phases using a 0.45 µm nylon membrane. We degasified them with an ultrasonic bath for 30 min. We set the column temperature at 25 °C, used the 254 nm UV detector, and established the flow of the mobile phase, injection volume, and run time as 0.2–0.8 mL/min, at 8 µL, and 35 min, respectively. To wash the piston seals, we used MeOH : H_2_O (60 : 50). To generate the calibration curves, we used standards for salicylic acid (SA), 4-hydroxybenzoic acid (4-HBA), chlorogenic acid (CGA), and quercetin (QUE) (Sigma-Aldrich). We generated the following calibration curves: *y*_i_ = 1109.4*x*_i_ + 481.67, *y*_i_ = 296.01*x*_i_ + 133.74, *y*_i_ = 551.41*x*_i_ + 263.64, and *y*_i_ = 919.96*x*_i_ + 201.64; here, *y*_i_ represents the area below the absorbance curve, *x*_i_ represents the concentration of the secondary metabolite, and *i* = 1, 2, 3, and 4 for 4-HBA, CGA, QUE, and SA, respectively. SA was not present in any of the samples tested (Table S1 online^31^).

We used a logistic regression model to test the effect of the sexual form, month of study, cladode age category, cladode size, the number of cladodes above a given cladode, and the cladode order above the soil level, on the probability of detecting secondary metabolites in the cladodes. Since the latter data were ordinal, the sexual form and month were considered as discrete variables and treated the other traits as continuous variables^60^. We applied the generalized linear mixed model (GLMM) with a logit link function [ln(*P*/(1-*P*)], where *P* indicated the probability of detecting a given metabolite, binomial response distribution, maximum likelihood estimation technique, Newton–Raphson optimisation algorithm, and Person Chi-Square/df fit criterion. We used the GLIMMIX procedure in SAS statistical software^61^ (Methods S1).

We used generalized linear models (GLMs) in R^47^ to determine the relationship between cladode length, width, thickness, months, age, cladode order from the soil, and cladodes above a given cladode, and the concentrations of the different secondary metabolites. Since many concentrations were null, we analysed only the positive concentrations (Methods S1).

### Comparison of damage between female and hermaphrodite individuals

We used the same plants as those used for relative growth rate analysis. We analysed the extent of damage caused by herbivorous insects on both sexes of *O. robusta* from March–June 2017. We selected two branches, one with flowers and the other with cladodes, from each plant. We estimated two types of damage caused by herbivores using image analysis, to determine the total percentage of tissue removed and other types of damage, such as scars or necrosis. We acquired photographs of one randomly selected face of each structure, using a Nikon D3200 with an AF-S DX NIKKOR 18–55 mm f/3.5–5.6G VR lens (Nikon Corporation, Tokyo, Japan) mounted on a tripod, using a 1-cm piece of millimetre paper as a reference for size. We analysed all images using ImageJ^62^ to estimate the total proportion of damaged areas.

We analysed data on herbivore damage and other damages using a GLM procedure with the Gaussian error distribution and identity link function^47^ in R. The response variables were the logit transformed proportion of damage (ln[*P*/(1-*P*)]), where *P* represents the proportion of tissue damaged. In our statistical models, the transformation improved the distribution of residuals. The explanatory variables were sex, type of structure, and their interactions. We performed partial regressions using the *ggeffects* package in R^48^.

### Comparison of the occurrence/concentrations of secondary metabolites between younger and older vegetative tissues

We named the oldest cladodes (closest to the soil) as ‘first-order cladodes,’ those growing on the oldest cladodes as ‘second-order cladodes’ etc. We selected each plant branch with the largest number of cladodes. We measured the length, width, and thickness of each cladode. We sampled vegetative tissues from plants belonging to each of the eight groups; the first group on the 10th March, the second group on the 12th April and so on, through the 10th May, 14th June, 12th July, 10th August, 13th September, and 11th October 2014. We measured the length and width of each cladode to the nearest 0.5 cm, using a measuring tape, and their thickness to the nearest 0.01 mm, using a calliper. We conducted the latter measurement in the apical part of the cladodes in the case of apical cladodes, or at the point of ramification of the daughter cladode when it grew on its apex.

During eight years of observations prior to the commencement of this study, we observed that the age of the cladodes in the studied zone could be estimated by examining the following colour patterns of their spines: 1—yellowish, 2—yellow, white base, 3—white-yellowish, 4—white, 5—greyish, 6—black, with ‘1’ being the youngest, and ‘6’ being the oldest. We assigned each cladode to one of the classes. We used the HPLC procedure described by Janczur and González Camarena^59^ to determine the concentrations of different secondary metabolites in the plant tissues.

To test whether different estimators of cladode age were parallel (to test whether younger cladodes were mostly apical, and thus bore fewer cladodes above), we examined the relationship between the cladode order from the soil or cladode number above a given cladode and cladode age, using ordinary least squares regression (OLS). We used a numerical algorithm applied to the SMATR software for R^63^. We included a test for the determination of the effects of cladode age estimators on the SMSs occurrence/concentration in the same GLM models, as described in the previous section.

### Trade-off between investments in defence, growth, and reproduction

We tested the relationship between cladode length and cladode order or cladode age to determine whether cladode size was parallel to cladode age. We performed OLS analysis and slope comparison between sexual forms using the Wald test (*W*_T_—test statistic) and tested the significance of differences between the intercepts. We used a numerical algorithm applied in the SMATR software^63^. To estimate the relative investment in growth and reproduction, we counted the number of flower and cladode buds on parental cladodes of the same plants used in the study performed by Sandoval and Janczur (Dataset online^29^). We used generalized linear models in R, with a negative binomial error distribution and log link function^47,55,56^, to test the effects of sexual form on the average number of flower and cladode buds. Significant differences between the number of flowers and cladodes for certain sexual forms implies a higher relative reproductive investment.

We used the same method of quantification for the standardized major axis and GLM models for intersexual comparisons, as described in the previous Sect. ^59^. For example, larger relative allocations for reproduction and secondary metabolites together with lower allocation to growth in one sexual form, compared to lower allocations for reproduction and secondary metabolites, and higher allocations for growth in the other sexual form imply that the production of secondary metabolites does not compete with either growth or reproduction; rather, growth competes with reproduction, and allocation to the production of secondary metabolites is an outcome of the gain in terms of fitness from such an allocation.

### Effects of the existence of trade-offs between different secondary metabolites on the predictions of the plant defence hypothesis

We used ordinary least squares regression (OLS), coefficient of determination, and *t*-tests to determine the existence of possible trade-offs in the proportion of cladodes harbouring different secondary metabolites. We performed the *t*-test to determine the significance of correlation between cladode order and cladode age^64^.

### Ethics statement

This research did not involve any human or animal measurements. We obtained permission from the head of the Singuilucan municipality, State of Hidalgo, Mexico, to conduct research activities at the selected sites of the municipality. The owners of the lands permitted us to conduct the study and were informed of the permission granted by the municipality. MKJ obtained a permit (09,448/14) from the Ministry of Environment and Natural Resources of the United States of Mexico (SEMARNAT), which stated that no permission is necessary to conduct field studies on plants belonging to the genus *Opuntia*. The study site was not considered to be a protected area^65^, and *O. robusta* was not considered to be an endangered species^66^. During this study, we did not affect or involve any endangered species. As we did not sample all plants, we did not deposit specimens in a public herbarium. No plant was killed or severely damaged as a result of our research activity; the plant material used for this study was sampled at a limited scale, and therefore, the sampling presented with negligible effects on the functions of the broader ecosystem. All the methods were carried out in accordance to relevant guidelines and regulations.

## Supplementary Information

Supplementary Information.

## Data Availability

Data sets can be accessed via the following links: https://doi.org/10.7910/DVN/LERCFK, https://doi.org/10.7910/DVN/QN3OUA.
