# Supplementary material for: Impact of the female and hermaphrodite forms of Opuntia robusta on the plant defence hypothesis
Source: Sci Rep. 2021 Jun 8;11:12063. doi: 10.1038/s41598-021-91524-5 (PMC8187663; doi:10.1038/s41598-021-91524-5)
Supplement: Supplementary file 1 — Supplementary Information. [file 41598_2021_91524_MOESM1_ESM.pdf]

Impact of the female and hermaphrodite forms of *Opuntia robusta* on the plant defence hypothesis

Mariusz Krzysztof Janczur[1,2*], Emilio González-Camarena[1], Hector Javier Leon-Solano[1,2], Mario Alberto Sandoval Molina[1,3], Bartosz Jenner[4]

[1]*Research Group in Ecology and Evolutionary Biology, Department of Natural Sciences, Autonomous University of the State of Mexico, México, Carretera Toluca-Tlachaloya, km 18, Cerrillo Piedras Blancas, CP 50200 Toluca, Estado de México, México*

email: majmx@interia.pl , phone: +52 722 296 5554 ext. 191

[2]*Graduate Program in Agricultural Sciences and Natural Resources (PCARN), Autonomous University of the State of Mexico, México, Carretera Toluca-Tlachaloya, km 18, Cerrillo Piedras Blancas, CP 50200 Toluca, Estado de México, México*

[3]*Instituto de Ecología A.C., Red de Ecología Funcional. Carretera Antigua a Coatepec 351, El Haya, Xalapa, Veracruz 91070, México.*

[4]*Actelion Pharmaceuticals Ltd, Gewerbestrasse 16, CH-4123 Allschwil, Switzerland*

[*]Mariusz Krzysztof Janczur ORCID: https://orcid.org/0000-0002-3886-6710

This document file includes:

Supplementary Information S1
Supplementary Results S1 and S2
Supplementary Tables S1 to S6
Supplementary Figures S1 to S6
Supplementary Discussion S1
Legends for Supplementary Tables S1 to S9 online (https://doi.org/10.7910/DVN/LERCFK/BI2OSB)
Supplementary References

Supplementary Information S1

Natural history of *Opuntia robusta*

Opuntia robusta is a plant form Cactaceae family. A most conspicuous characteristic of the plants from this genus is the presence of cladodia that are photosynthetic flattened branches or portion of a stem that functions as or resembles a leaf. Cladodes produce on their surfaces and borders groups of areoles that are small bumps out of which grow clusters of spines. This species is almost endemic to Mexico however, it is widely distributed in arid and semi-arid regions of the country, in cactus-dominated scrubland), but also in *Pinus* and *Quercus* forests and grasslands of the states of Aguascalientes, Chihuahua, Coahuila, Durango, Guanajuato, Hidalgo, Jalisco, Mexico, Michoacán, Morelos, Nuevo León, Puebla, Querétaro, San Luis Potosí, Sonora, Tamaulipas, Tlaxcala, Veracruz, Zacatecas, and México City [1,2]. At the study site the blossoming begins in late February and ends in late July. Most of the pollinators of *O. robusta* are bees from Anthophoridae, Megachilidae, Halictidae, Apidae and Andrenidae families [3,4].

Young cladodes develop areoles with modified and secretory spines as EFNs only active during the early growth phase. EFNs are non-vascularized structures, with no stomata, that consist of a basal meristematic tissue, a middle elongation region, and an apical secretory cone formed by large globular epidermal cells, containing nectar [5].

Secondary compounds found in *Opuntia robusta*.

At least 28 secondary compounds have been already described in *Opuntia* genus (mainly, *O. ficus-indica*): **quercetin (QUE)**, kaempferol, quercetin 3-*O*-methyl ether, 2,3-dihydrokaempferol, isorhamnetin 3-*O*-glucoside, 2,3- dihydroquercetin, coumaric acid, kaempferol 7-*O*-glucoside, ferulic acid, isorhamnetin 3-*O*-neohesperidoside,

isorhamnetin 3-*O*-rutinosyl- 4'-*O*-ß-D-glucoside, isorhamnetin 3-*O*-(2,6-dirhamnosyl)glucoside, zataroside-A, n-butyl-ß-D-fructopyr-anoside, 4-*O*-glucosyl-mapic acid, isorhamnetin-3-*O*-(6''-*O*-*E*-feruloyl)neohesperidoside, and (6R)-9,10-dihydroxy-4,7-megastigmadien-3-one-9-*O*-ß-D-glucopyranoside, gallic acid, epicatechin gallate, vanillic acid, chlorogenic acid (CGA), procyanidin B2, epicatechin, epigallocatechin, catechin gallate, sinapic and benzoic acids, hyperoside, isoquercetin, and rutin [6,7]. In *O. robusta*, Guevara-Figueroa, et al.[8] found gallic, coumaric, 3,4-dihydroxy-benzoic, 4-hydroxy benzoic (4-HBA), ferulic, salicylic acids (SAL), iso-quercitrin, isorharmnetin 3-O-glucoside, nicotiflorin, rutin, and narcissin in cladodes, and Gonzalez-Ponce, et al.[9] found QUE in fruits.

Defensive function of secondary metabolites found in *Opuntia robusta*

There exists only a limited amount of data concerning secondary metabolites in cladodes of *O. robusta*. Guevara-Figueroa, et al.[8] found 10 different secondary metabolites in cladodes of this species, among them **4-hydroxybenzoic acid** (4-HBA) and **salicylic acid** (SA; **2-hydroxybenzoic acid**). Gonzalez-Ponce, et al.[9] found **quercetin** (QUE) in fruits. As far as we know, there is no study confirming the presence of **chlorogenic acid** (CGA) in *O. robusta*. Neither do studies exist concerning the ecological aspects of secondary metabolites in this species besides our previous study [10]. However, the defensive function of some of the metabolites found either in *O. robusta* or in other *Opuntia* species was confirmed in other plant species, i.e. CGA combated arthropods [11-17], microbes [18], and fungi [19]; 4-HBA combated microbes [20]; QUE deterred arthropods [17], nematodes [21] and fungi [22,23]. Though SA is known as a universal phytohormone that mediates induced plant

defense [24-27], strangely nothing is known about the possible inductive function of this metabolite in response to herbivory, either in *Opuntia* genus.

Supplementary Methods

Do female individuals reveal a higher incidence/concentration of secondary metabolites than hermaphrodite individuals?

The number of possible interactions between sexual form and other variables was very high (2 × 6 × 8 × 5 × 5, for sex, cladode age, months, cladode order and the number of cladodes above a given cladode, respectively). The numbers of cladodes were unbalanced and low for most of the interactions between particular combinations of levels of the independent variables since most of the cladodes did not contain secondary metabolites. Considering this, when we analysed the effect of a given independent variable we pooled the data for the other independent variables. We performed this analysis from a "cladode point-of-view." That is, we studied the response of cladodes in producing secondary metabolites to cladode state (sexual form, size, cladode age) and to month of the year which, in turn, depended on meteorological factors. We used a logistic regression model to test the effect of the sexual form, month of study, cladode age category, cladode size, the number of cladodes above a given cladode, and the cladode order above soil level, on the probability of detection of cladodes bearing a given secondary metabolite. Since the latter data were ordinal, we treated the sexual form and month as discrete variables, and treated the other traits as continuous variables [28]. We applied the generalized linear mixed model (GLMM) with logit link function ($\ln(P/(1-P))$, where P – probability of detection of a given metabolite), binomial response distribution, maximum likelihood estimation technique, Newton-Raphson optimization

algorithm, and Person Chi-Square/df fit criterion. We used GLIMMIX procedure of the SAS statistical software [29].

To analyse how concentrations of the different substances were related to cladode length, width, thickness, months, age, cladodes order from soil, and cladodes above a given cladode, we used generalized linear models (GLMs) created in R [30]. Since many concentrations were null, we analysed only the positive concentrations. The full model contained the interaction between each individual variable and sex. For each response variable, we used different error distribution and link functions that gave the best fit of the models. To analyse the effect of months and age on 4-HBA concentrations, we used the Gamma error distribution (GED) and the inverse link function. For the effect of months on CGA concentrations, we used GED and log link functions. For the effect of cladode age on CGA concentrations, we used GED and inverse link functions. For the effect of months on QUE concentration, we used GED and log link functions. For the effect of cladode age on QUE concentration, we used GED and inverse link function. For the models that related substances with cladode length, width and thickness, we used different models: for 4-HBA concentrations we used a GED with square root link function. For CGA concentrations, we used GED and identity link function; for QUE concentrations, we used GED and inverse link function. To test the effect of cladodes order from soil on the concentrations of the three substances, we used GED and square root link function. To test the effect of the number of cladodes above a given cladode on the concentrations of the three substances, we used GED and inverse link function.

We checked the goodness of fit of the linear models (GLM or GLMM) by plotting the standardized residuals against fitted values and by normal QQ-plots, and revised the

assumptions of the homoscedasticity, proper distribution used, and independence. We chose the best GLM model used in this study according to the lowest AIC [31] following the recommendations of Zuur, et al.[32].

Supplementary Results S1

The effect of meteorological factors on the proportion of cladodes bearing secondary metabolites

Total per-month precipitation was higher at the beginning of the rainy season (July) and decayed in October. Global radiation was higher in dry than in rainy season. Relative humidity increased during the rainy season. Maximum temperature decreased almost monotonically through the season. Minimum temperature did not show a clear tendency. Potential evapotranspiration decreased during season. Several meteorological variables were correlated, i.e. average per-month relative humidity depended negatively on the maximum temperature (86% as explained by the model), but not on the minimum temperature. Per-month minimum temperature did not depend on wind speed, but wind speed increased with increasing maximum temperature. Potential per-day evapotranspiration was positively and significantly related to maximum, minimum, and average temperatures. The relationships among the other meteorological variables were rather obvious. The average per-month and per-day values of maximum temperature, average temperature, mean wind speed, relative humidity and cumulative values of evapotranspiration of reference were significantly correlated (Table S2; Figure S1; Janczur, et al.[33]).

Table S1 Generalized linear model and post-hoc contrasts for the effects of plant sexual form and structure type (cladodes or flowers), and cumulative proportion of damage on the relative growth rate of the vegetative (cladodes) and reproductive (flowers) biomass of Opuntia robusta. The explicative variable was the cumulative proportion of damage for each plant structure transformed with logit. Estimates – model parameter estimates; SE – standard error of the model effect estimator; Statistic – test statistics; AIC – Akaike information criterion. The values of P for effects significant at P ≤ 0.05 are marked with bold text.

	Relative growth rate			
Predictors	Estimates	SE	Statistic	*P*
(Intercept)	0.099	0.027	3.650	**< 0.001**
sex [Hermaphrodite]	0.046	0.055	0.833	0.41
type [Flower]	-0.065	0.024	-2.688	0.01
herb.total.logit.last	-0.004	0.003	-1.237	0.22
sex [Hermaphrodite] × type [Flower]	0.052	0.063	0.830	0.41
Observations	129			
R^2 Nagelkerke	0.160			
AIC	-220.674			
log-Likelihood	116.337			

Post-hoc contrasts				
Contrast	Estimates	SE	Statistic	*P*
Cladodes: Female − Hermaphrodite	-0.04	0.05	-0.833	0.40
Flowers: Female − Hermaphrodite	-0.098	0.03	-3.258	**0.001**

Table S2. Relationship among meteorological parameters. A) Percentage of the variation ($R^2 \times 100$) explained by the relationship within the meteorological data, and between the meteorological data and the proportions of cladodes bearing 4-HBA, CGA, and QUE (above diagonal) and probability *P* of the respective correlation coefficient (below diagonal). The sign of the percentage of variation denotes the sign of the correlation coefficient. Meteorological data are averages for sampling days or cumulative from the beginning through the sampling day (TP, REVT, PEVT) for March through October 2014. B) Percentage of variation explained by the relationship among per-month and per-day average or cumulative values. (-) – negative relationships, TP – total precipitation [mm], MAXT – maximum temperature [°c], MINT – minimum temperature [°C], AVERT – average temperature [°C], MAXWS – maximum wind speed [km/h], MEANWS – mean wind speed [km/h], GR – global radiation [$W(m^2)^{-1}$], RH – relative humidity [%], REVT – evapotranspiration of reference [mm], PEVT – potential evapotranspiration [mm]. Results significant at 0.05 are marked with bold.

a)

P \ R^2	**TP**	**MaxT**	**MinT**	**AverT**	**MaxWS**	**MeanWS**	**GR**	**RH**	**REVT**	**PEVT**
**TP**		-35.0	21.3	-13.6	-7.7	**-55.9**	-28.2	**58.0**	**-50.6**	**-47.6**
**MaxT**	0.1		0.0	**83.8**	**58.2**	**48.4**	**75.3**	**-85.9**	**93.1**	**81.5**
**MinT**	0.24	0.96		14.1	-0.2	-3.9	-16.3	9.3	-5.5	-19.3
**AverT**	0.36	**0.001**	0.35		**47.6**	27.8	41.3	**-55.1**	**65.0**	43.6
**MaxWS**	0.5	**0.02**	0.92	**0.048**		14.2	39.3	**-48.6**	**47.4**	**48.4**
**MeanWS**	**0.03**	**<0.05**	0.63	0.17	0.35		33.7	**-52.7**	**48.3**	44.7
**GR**	0.16	**<0.01**	0.3	0.07	0.08	0.12		**-75.6**	**83.4**	**92.5**
**RH**	**0.02**	**<0.01**	0.45	**0.03**	**<0.05**	**0.03**	**<0.01**		**-96.9**	**-90.4**
**REVT**	**0.04**	**<0.01**	0.57	**0.01**	**<0.05**	**<0.05**	**<0.01**	**<0.001**		**92.5**
**PEVT**	**<0.05**	**<0.01**	0.27	0.06	**<0.05**	0.06	**<0.001**	**<0.001**	**<0.001**	

b)

	**TP**	**MaxT**	**MinT**	**AverT**	**Max WS**	**Mean WS**	**GR**	**RH**	**REVT**	**PEVT**
R^2	42.99	49.73	20.49	70.14	7.28	56.01	13.60	73.63	67.57	36.82
P	0.066	**0.041**	0.249	**0.006**	0.512	**0.025**	0.360	**0.003**	**0.008**	0.098

Figure S1. Dynamics of average monthly total precipitation, global radiation, relative humidity, maximum air temperature, minimum air temperature, and potential evapotranspiration, from March through October 2014

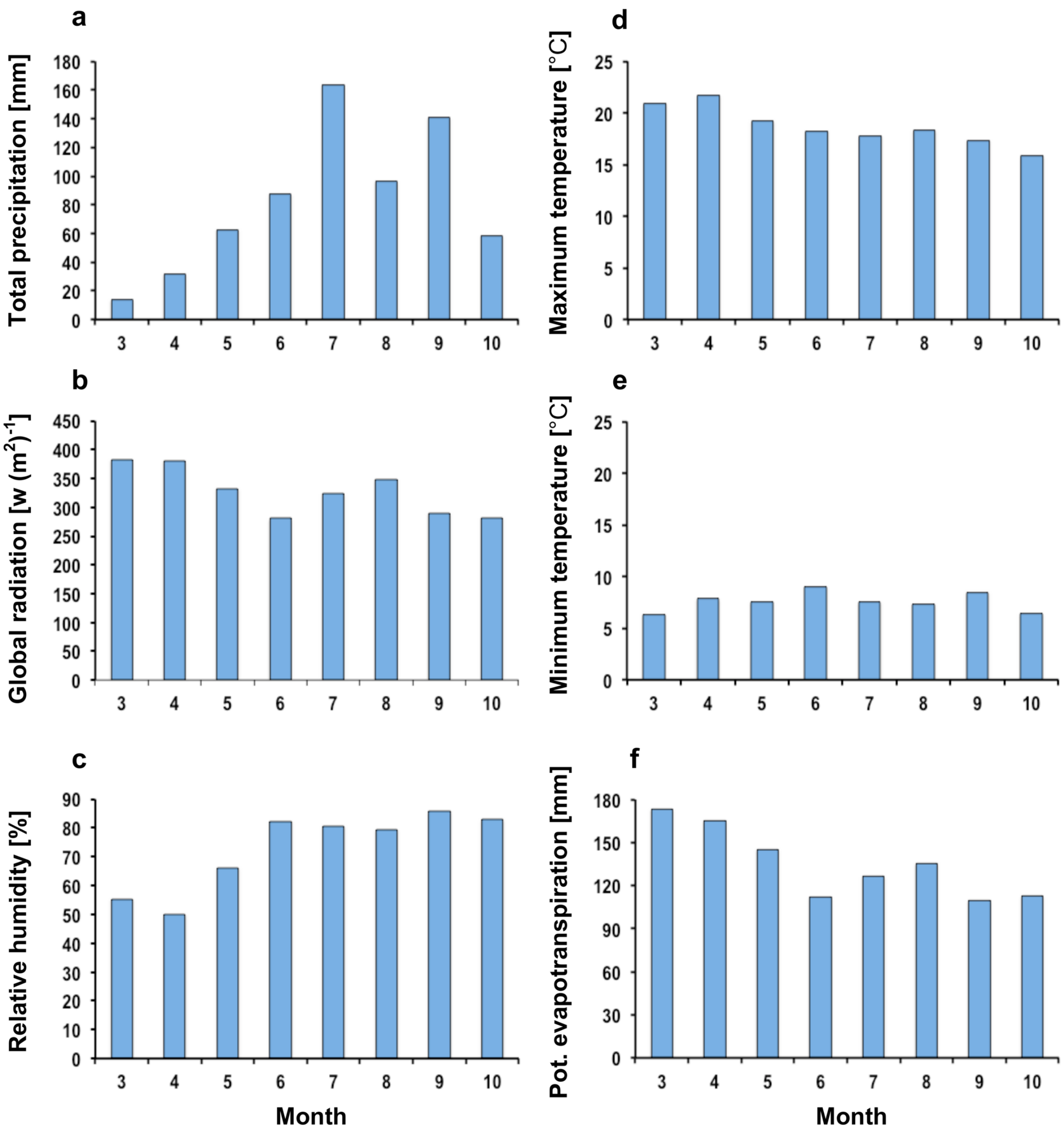

Table S3. Model statistics and post-hoc contrasts for the effect of sexual form on the number of flowers and cladodes produced during the growing/reproductive season of *Opuntia robusta* in Central Mexico. We fitted a generalized linear model (GLM) with negative binomial error distribution. SE – standard error of the model effect estimator; Statistic – test statistics; df – degree of freedom; Sex [Herm] - the effect of hermaphrodite sexual form; AIC – Akaike information criterion. The values of *P* for effects significant at $P \leq 0.05$ are marked with bold text.

	**Number of cladode or flower buds**			
Predictors	Occurrence Rate Ratios	SE	Statistic	*P*
(Intercept)	3.875	0.258	5.259	**< 0.001**
sex [Hermaphrodite]	0.568	0.460	-1.230	0.219
type [Flower]	3.908	0.339	4.024	**< 0.001**
sex [Herm] × type [Flower]	0.509	0.568	-1.189	0.234
Observations	28			
R^2 Nagelkerke	0.781			
AIC	152.537			

**Contrasts between sex and type**						
Contrast	Structure type	ratio	SE	df	z ratio	*P*
Female / Hermaphrodite	Cladode	1.761	0.811	Inf	1.23	0.219
Female / Hermaphrodite	Flower	3.461	1.153	Inf	3.73	**< 0.001**
**Contrasts between sex and type**						
Contrast	Sexual form	ratio	SE	df	z ratio	*P*
Cladode / Flower	Female	0.256	0.087	Inf	-4.02	**< 0.001**
Cladode / Flower	Hermaphrodite	0.503	0.229	Inf	-1.51	0.132

Table S4. Model statistics and post-hoc contrasts for the effects of sexual form (female, hermaphrodite) on the number of fruits eaten, fruit volume, fresh biomass, and fruit density for *Opuntia robusta* in central Mexico. We fitted a generalized linear model (GLM): for fruit number with negative binomial error distribution, and for volume, fresh biomass, and density, with Gaussian distribution and a log link function. Inc. – Incidence rate ratio; SE – Standard error of the model estimator; Stat – test statistics; Est – model parameter estimator; df – degree of freedom; Sex [Her] - the effect of hermaphrodite sexual form; AIC – Akaike information criterion. The values of *P* for effects significant at $P \leq 0.05$ are marked with bold text.

	**Fruits eaten**				**Volume**				**Fresh biomass**				**Biomass /volume**			
Predictors	Inc.	SE	Stat	*P*	Est	SE	Stat	*P*	Est	SE	Stat	*P*	Est	SE	Stat	*P*
(Intercept)	7.39	0.36	5.5	**<0.001**	54.5	2.66	20.5	**<0.001**	105.5	5.34	19.7	**<0.001**	1.91	0.036	53.5	**<0.001**
Sex [Her]	0.441	0.42	-1.97	**0.05**	8.7	3.09	2.82	**0.005**	15.7	6.22	2.5	**0.012**	0.01	0.042	0.26	0.796
Obs.	60				198				198				198			
AIC	299.069				1736.967				2013.790				30.444			

**Contrasts per sex, fruits eaten**					
Contrast	Ratio	SE	df	t ratio	*P*
Female / Hermaphrodite	2.268	0.942	57	1.973	0.053
**Contrast between sexual forms, fruit volume**					
Contrast	Estimate	SE	df	t ratio	*P*
Female − Hermaphrodite	-8.716	3.092	195	-2.819	**0.005**
**Contrast between sexual forms, fresh biomass [g]**					
Female − Hermaphrodite	-15.670	6.221	195	-2.519	**0.013**
**Contrasts between sexual forms, fruit density [g × cm^{-3}]**					
Female − Hermaphrodite	-0.011	0.042	195	-0.259	0.796

Table S5. Basic statistics for the percentage of cladodes bearing 4-hybrobenzoic acid (4-HBA), chlorogenic acid (CGA), or quercetin (QUE) (a) and their concentrations in cladodes (b), for sexual form, month of sampling, cladode age class, cladode order, and number of cladodes growing above a given cladode. Cladode age classes are based on the spine color: 1 – yellowish, 2 – yellow, white base, 3 – white yellowish, 4 – white, 5 – grayish, 6 – black, being "1" the youngest, and "6", the oldest age class. Tot – total number of cladodes, N – number of cladodes bearing a metabolite, fraction - fraction of cladodes bearing a metabolite, ± CI – 95% confidence interval for fraction. Two outliers were removed from the analysis of concentrations of the secondary compounds.

a)

Trait analysed	Tot	N 4-HBA	Fraction 4-HBA	-CI	+CI	N CGA	Fraction CGA	-CI	+CI	N QUE	Fraction QUE	-CI	+CI
Sex													
**F**	99	39	39.4%	29.7%	49.7%	20	20.2%	12.8%	29.5%	29	29.3%	20.6%	39.3%
**H**	283	43	15.2%	11.2%	19.9%	37	13.1%	9.4%	17.6%	20	7.1%	4.4%	10.7%
Month of sampling													
**March**	48	14	29.2%	17.0%	44.1%	6	12.5%	4.7%	25.2%	1	2.1%	0.1%	11.1%
**April**	48	10	20.8%	10.5%	35.0%	7	14.6%	6.1%	27.8%	13	27.1%	15.3%	41.8%
**May**	49	11	22.4%	11.8%	36.6%	10	20.4%	10.2%	34.3%	4	8.2%	2.3%	19.6%
**June**	46	14	30.4%	17.7%	45.8%	5	10.9%	3.6%	23.6%	6	13.0%	4.9%	26.3%
**July**	46	7	15.2%	6.3%	28.9%	6	13.0%	4.9%	26.3%	4	8.7%	2.4%	20.8%
**August**	46	6	13.0%	4.9%	26.3%	10	21.7%	10.9%	36.4%	6	13.0%	4.9%	26.3%
**September**	48	4	8.3%	2.3%	20.0%	4	8.3%	2.3%	20.0%	2	4.2%	0.5%	14.3%
**October**	51	16	31.4%	19.1%	45.9%	9	17.6%	8.4%	30.9%	13	25.5%	14.3%	39.6%
Cladode age class													
**1**	18	3	16.7%	3.6%	41.4%	9	50.0%	26.0%	74.0%	0	0.0%	0.0%	18.5%
**2**	9	1	11.1%	0.3%	48.2%	6	66.7%	29.9%	92.5%	0	0.0%	0.0%	33.6%
**3**	158	41	25.9%	19.3%	33.5%	29	18.4%	12.7%	25.3%	23	14.6%	9.5%	21.0%
**4**	29	7	24.1%	10.3%	43.5%	5	17.2%	5.8%	35.8%	5	17.2%	5.8%	35.8%
**5**	72	10	13.9%	6.9%	24.1%	6	8.3%	3.1%	17.3%	6	8.3%	3.1%	17.3%
**6**	96	20	20.8%	13.2%	30.3%	2	2.1%	0.3%	7.3%	15	15.6%	9.0%	24.5%
Cladode order													
**1**	96	20	20.8%	13.2%	30.3%	2	2.1%	0.3%	7.3%	15	15.6%	9.0%	24.5%
**2**	96	14	14.6%	8.2%	23.3%	9	9.4%	4.4%	17.1%	11	11.5%	5.9%	19.6%
**3**	96	19	19.8%	12.4%	29.2%	18	18.8%	11.5%	28.0%	13	13.5%	7.4%	22.0%
**4**	80	25	31.3%	21.3%	42.6%	20	25.0%	16.0%	35.9%	10	12.5%	6.2%	21.8%
**5**	14	4	28.6%	8.4%	58.1%	8	57.1%	28.9%	82.3%	0	0.0%	0.0%	23.2%
Number of cladodes above a given cladode													
**0**	97	27	27.8%	19.2%	37.9%	32	33.0%	23.8%	43.3%	6	6.2%	2.3%	13.0%
**1**	96	21	21.9%	14.1%	31.5%	18	18.8%	11.5%	28.0%	16	16.7%	9.8%	25.6%
**2**	97	19	19.6%	12.2%	28.9%	6	6.2%	2.3%	13.0%	12	12.4%	6.6%	20.6%
**3**	77	15	19.5%	11.3%	30.1%	1	1.3%	0.0%	7.0%	13	16.9%	9.3%	27.1%
**4**	15	0	0.0%	0.0%	21.8%	0	0.0%	0.0%	21.8%	2	13.3%	1.7%	40.5%

b)

Trait analysed	N 4-HBA	4-HBA	SD	CI	N CGA	CGA	SD	CI	N QUE	QUE	SD	CI
Sex												
**F**	37	3.344	1.856	0.619	20	9.9	5.568	2.606	28	5.748	3.848	1.492
**H**	43	4.022	2.088	0.643	37	10.839	5.931	1.977	20	3.61	1.254	0.587
Month of sampling												
**March**	13	4.671	2.594	1.568	6	8.685	4.615	4.843	1	2.65	NA	NA
**April**	9	3.372	1.623	1.247	7	8.15	2.288	2.116	12	4.998	3.767	2.393
**May**	11	4.902	1.734	1.165	10	9.86	4.676	3.345	4	3.652	0.36	0.573
**June**	14	2.949	1.802	1.041	5	15.5	4.903	6.088	6	6.477	4.867	5.108
**July**	7	2.434	1.563	1.446	6	8.758	2.867	3.008	4	2.438	0.305	0.486
**August**	6	3.172	1.856	1.948	10	14.261	8.846	6.328	6	4.778	3.17	3.327
**September**	4	4.515	2.246	3.574	4	8.987	4.513	7.182	2	6.825	5.961	53.557
**October**	16	3.516	1.674	0.892	9	9.189	5.676	4.363	13	4.999	2.307	1.394
Cladode age class												
**black**	19	3.135	1.665	0.802	2	11.785	2.157	19.377	14	4.483	2.544	1.469
**gray-white**	10	3.084	1.239	0.886	6	10.085	4.714	4.948	6	5.635	4.742	4.977
**white**	7	3.121	1.37	1.267	5	8.83	4.657	5.783	5	7.706	4.257	5.285
**white-yellowish**	40	4.212	2.343	0.749	29	11.201	6.562	2.496	23	4.263	2.694	1.165
**yellow-white base**	1	2.64	NA	NA	6	8.3	3.017	3.166				
**yellowish**	3	4.43	0.913	2.268	9	10.691	6.625	5.092				
Cladode order												
**1**	19	3.135	1.665	0.802	2	11.785	2.157	19.377	14	4.483	2.544	1.469
**2**	14	3.012	1.159	0.669	9	10.236	4.696	3.609	11	6.576	4.434	2.979
**3**	19	4.062	2.385	1.15	18	10.326	5.239	2.605	13	4.962	3.252	1.965
**4**	24	4.358	2.213	0.935	20	11.758	7.513	3.516	10	3.354	1.418	1.014
**5**	4	3.29	1.59	2.531	8	7.792	2.719	2.273				
Number of cladodes above a given cladode												
**0**	26	4.18	2.114	0.854	32	10.874	6.736	2.429	6	3.185	1.307	1.372
**1**	21	3.937	2.288	1.041	18	9.633	4.38	2.178	16	4.979	3.453	1.84
**2**	19	2.979	1.761	0.849	6	12.123	3.739	3.924	12	5.674	4.182	2.657
**3**	14	3.479	1.422	0.821	1	4.98	NA	NA	12	4.237	1.789	1.137
**4**									2	7.725	4.688	42.121

Table S6. Generalized mixed-effect model and post-hoc contrasts for the effects of plant sex (female, hermaphrodite) and structure type (cladodes or flowers) on the cumulative herbivory of vegetative (cladodes) and reproductive (flowers) biomass for *Opuntia robusta* in central Mexico. Estimates – model parameter estimates; SE – standard error of the model effect estimator; Statistic – test statistics; AIC – Akaike information criterion. The values of P for effects significant at $P \leq 0.05$ are marked with bold text.

	Cumulative herbivory			
Predictors	Estimates	SE	Statistic	*P*
(Intercept)	-6.296	0.455	-13.846	**< 0.001**
Hermaphrodites	-0.837	1.227	-0.682	0.495
Flowers	2.294	0.512	4.483	**< 0.001**
Hermaphrodites: Flowers	-4.326	1.373	-3.152	**0.002**
Observations	471			
Marginal R^2 / Conditional R^2	0.069 / 0.846			
AIC	2482.424			
log-Likelihood	-1233.212			

Post-hoc contrasts				
Contrast	Estimates	SE	Statistic	*P*
Cladodes: Female − Hermaphrodite	0.836	1.227	0.682	0.496
Flowers: Female − Hermaphrodite	5.163	0.614	8.402	**< 0.001**

Figure S2. Relationships between cladode traits. The relationships between cladode length and cladode order (a), or cladode age class (b), maintained the same slope for both sexual forms; hermaphrodite cladodes of all orders and age classes were larger than respective female cladodes. y1 – cladode order from soil (c) and y2 – number of cladodes above a given cladode (d), were a good estimators of cladode age (x). Regression equations and t test P on the subfigures refers the non-transformed data. To make the data continuous, we performed the significance test for ln transformed data: y1 = -0.8891x + 2.6069; R2 = 0.64; P < 0.0001 and y2 = 1.2644x - 1.1525; R2 = 0.63; P < 0.0001, for (c) and (d), respectively, for ln(cladode order from soils +1), ln(cladode levels above + 1.1), and ln(cladode age class + 1).

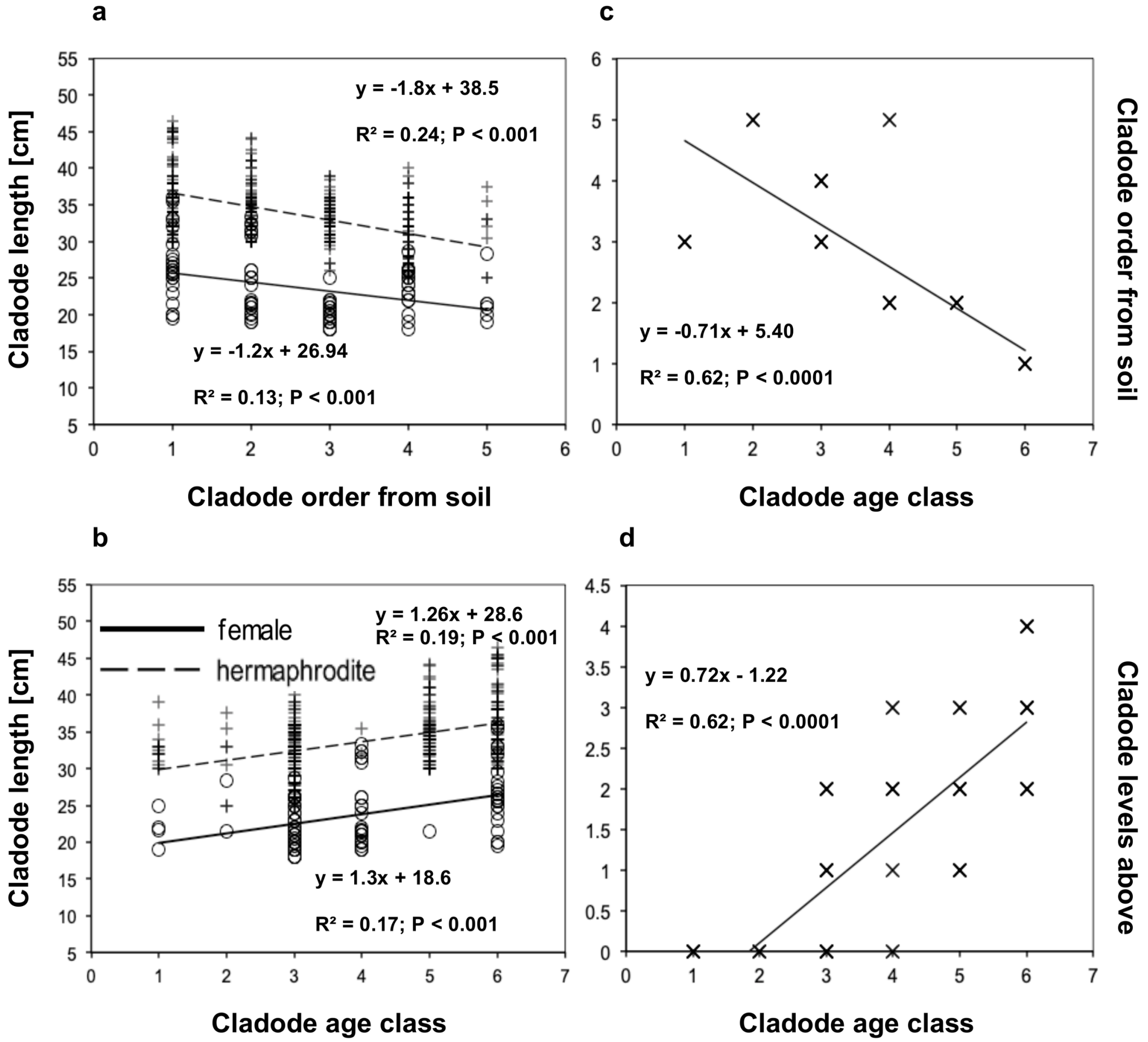

Supplementary Results S2

Dynamics of the secondary metabolites

We found a significant effect of month-of-year on the proportion of cladodes with 4-HBA: the proportion was significantly higher in March than in July, August and September; it was higher in May than in September; and it was higher in June than in July, August, and September. Finally, the proportion of cladodes with 4-HBA increased in October with respect to proportions found in July, August, and September (Figure S3 a). We did not find significant dynamics of the CGA (Figure S3 b). The proportion of cladodes with QUE was very low in March and increased significantly in April; this proportion decreased either significantly or nearly significantly again from May through September. Similarly, as in the case of 4-HBA, the proportion of cladodes with QUE increased in October, as compared to March, May, July and September (Figure S3 c; Table S5 a; Supplementary Table S1, S3, and S4 online [34]).

We did not find significant dynamics of the concentration of secondary metabolites. The effect of sexual form on the average concentration of 4-HBA and QUE was significant, but not so on the concentration of CGA (Figure S3 d, e and f). Additionally, there existed a significant interaction between sexual form and month. For most months, the concentration of 4-HBA was either higher in hermaphrodite cladodes or similar in both sexual forms, but, in August, the concentration was higher in female cladodes than hermaphroditic cladodes (Figure S3 d). For most months, the concentration of QUE was higher in female individuals than hermaphroditic individuals (Figure S3 f; Table S5 b; Supplementary Table S5 online [35]).

Figure S3. Dynamics of the proportion of cladodes bearing a – 4-hydroxybenzoic acid (4-HBA), b – chlorogenic acid (CGA), and c – quercetin (QUE), or of their concentrations (d, e, and f), from March through October 2014, for female and hermaphrodite cladodes. Female cladodes bore 4-HBA and QUE with higher probability than hermaphrodite cladodes. The probability of detection of CGA did not show significant dynamics. *P* – probability of adjustment in logistic regression model for pooled sexual forms. The concentrations of the four secondary metabolites did not show significant dynamics. We presented here box-and-whisker plots for each month and sexual form.

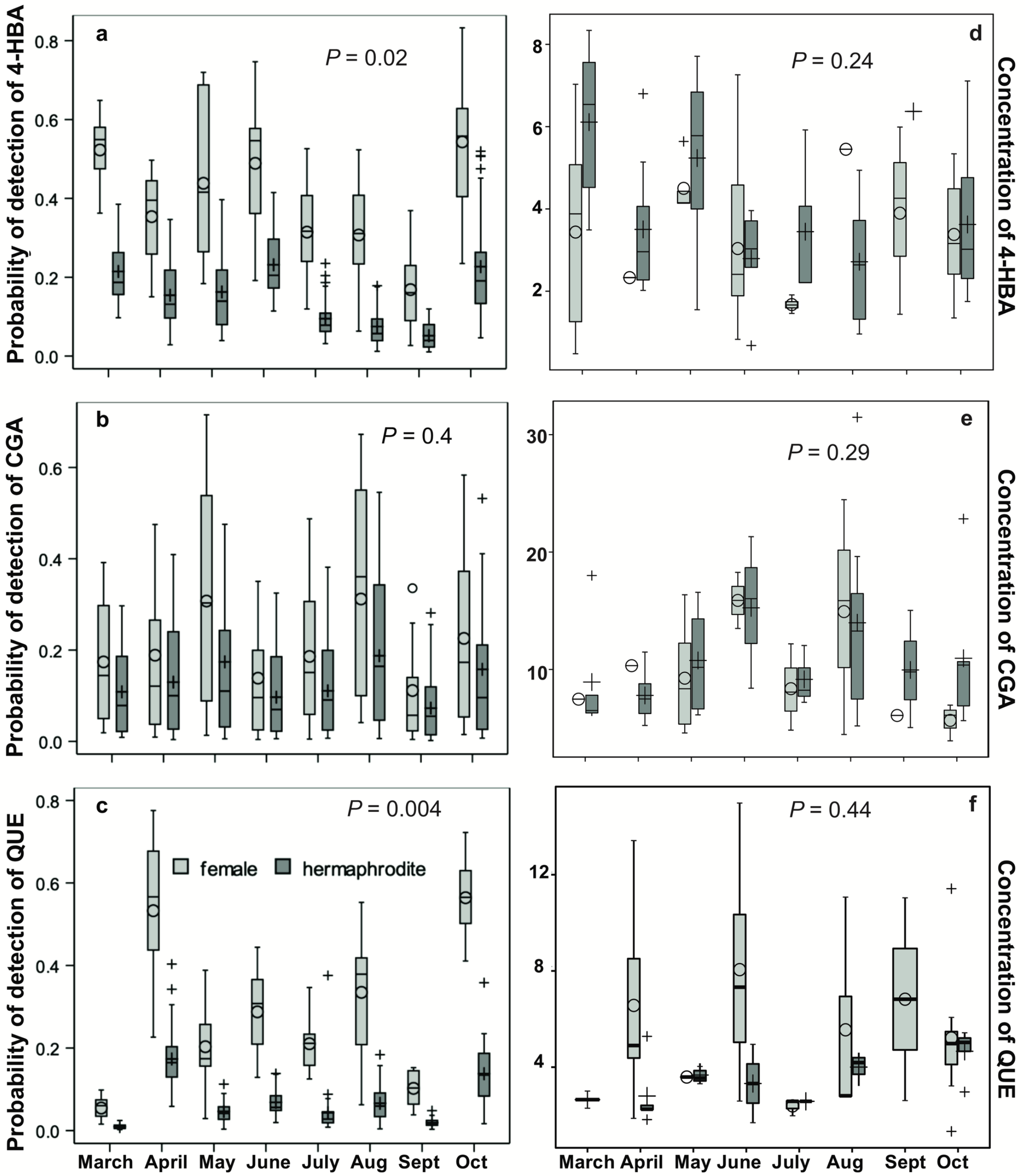

Figure S4. The effect of cladode age class on the concentration of a – 4-hydroxybenzoic acid (4-HBA), b – chlorogenic acid (CGA), and c – quercetin (QUE) in the vegetative tissues of female and hermaphrodite cladodes. The adjustment for the entire model was not significant for any of the three secondary metabolites however, it was significant for 4-HBA in hermaphrodite cladodes. *P* – probability of adjustment of the GLM model for a given sexual form. Cladode age classes are based on the spine colour: 1 – yellowish, 2 – yellow, white base, 3 – white yellowish, 4 – white, 5 – grayish, 6 – black, being “1” the youngest, and “6”, the oldest age class

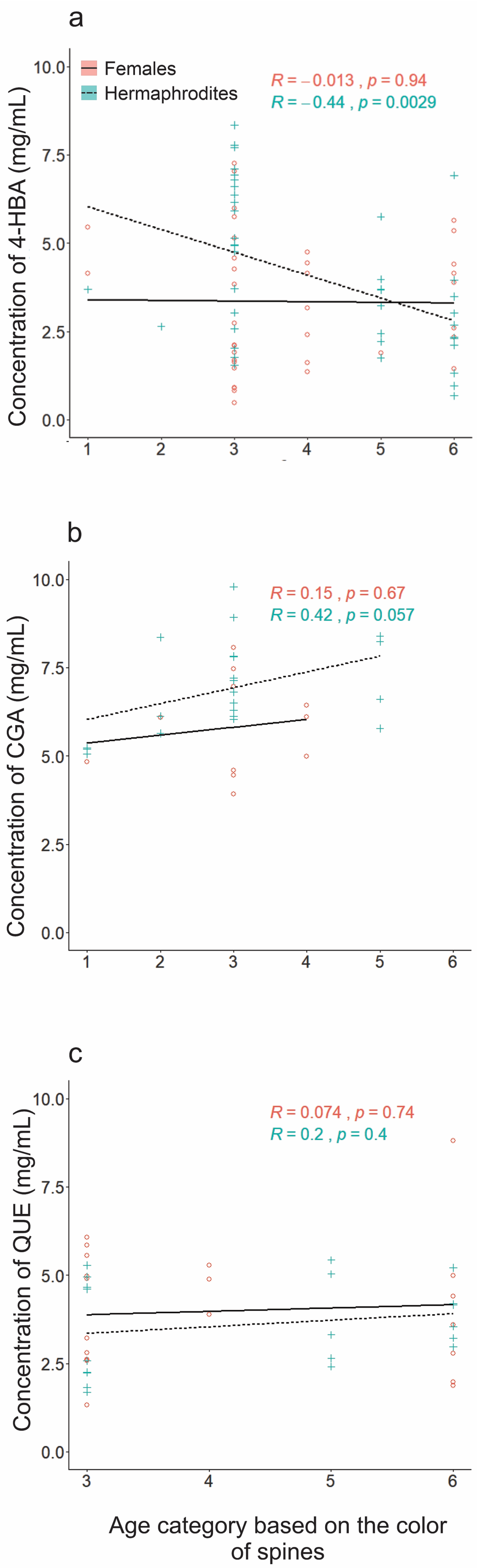
a
Females
Hermaphrodites
R = −0.013 , p = 0.94
R = −0.44 , p = 0.0029
Concentration of 4-HBA (mg/mL)
b
R = 0.15 , p = 0.67
R = 0.42 , p = 0.057
Concentration of CGA (mg/mL)
c
R = 0.074 , p = 0.74
R = 0.2 , p = 0.4
Concentration of QUE (mg/mL)
Age category based on the color of spines

Figure S5. The effect of cladode length (**a**, **b**, **c**), width (**d**, **e**, **f**) and thickness (**g**, **h**, **i**) on the concentration of 4-hydrobenzoic acid (4-HBA), chlorogenic acid (CGA), and quercetin (QUE) in the vegetative tissues of female and hermaphrodite cladodes. For hermaphrodites, the concentration of 4-HBA was significantly higher in younger cladodes. The concentration of 4-HBA was higher in hermaphrodite than in female cladodes. The relationship between 4-HBA concentration and hermaphrodite cladode length and width was significant and negative. The concentration of CGA was higher in wider hermaphrodite cladodes. The concentration of QUE was higher in female than in hermaphrodite cladodes, was lower in wider and higher in thicker hermaphrodite cladodes. *P* – probability of adjustment of the GLM model for a given sexual form

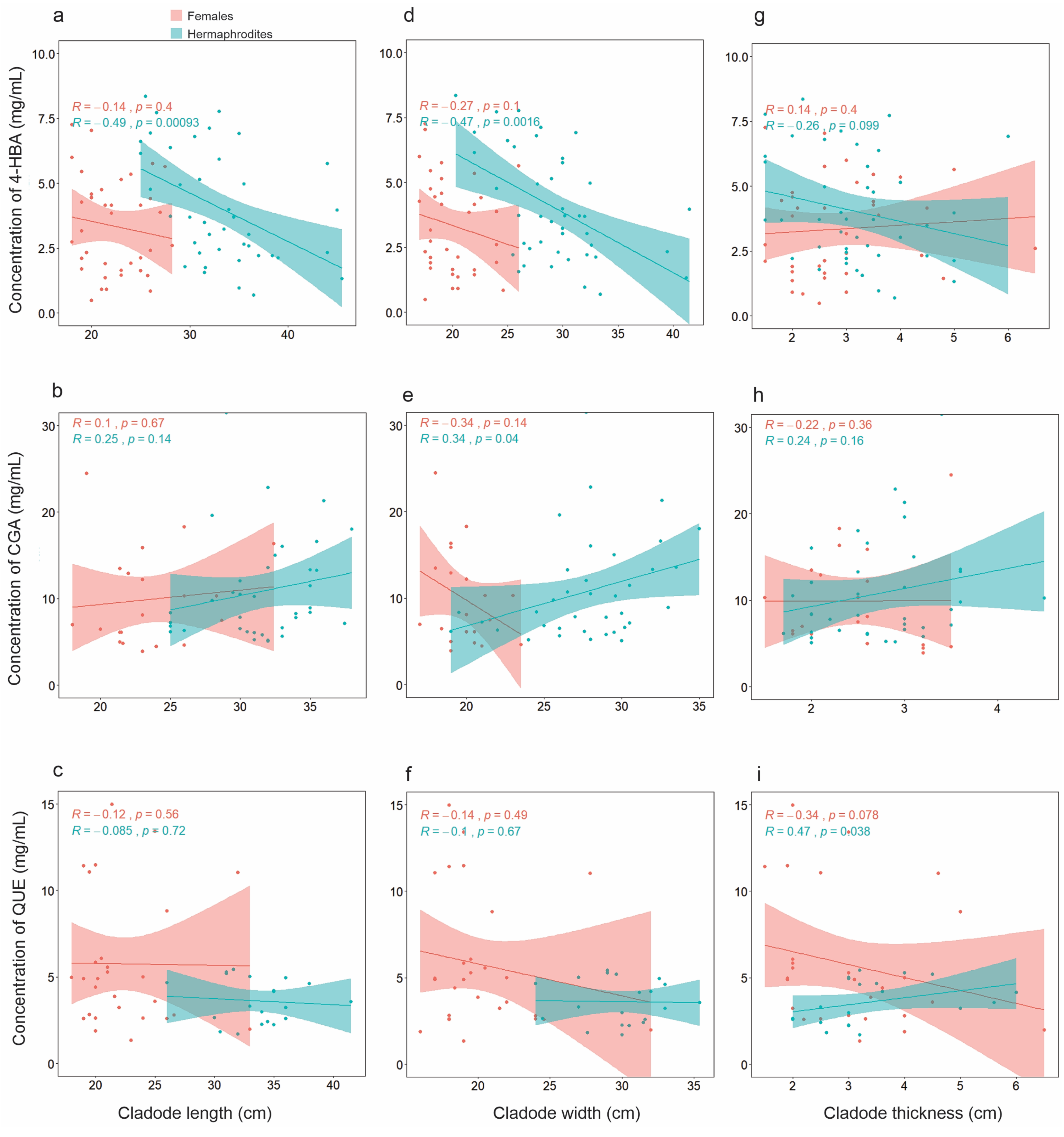

a
Females
Hermaphrodites
R = −0.14 , p = 0.4
R = −0.49 , p = 0.00093
d
R = −0.27 , p = 0.1
R = −0.47 , p = 0.0016
g
R = 0.14 , p = 0.4
R = −0.26 , p = 0.099
Concentration of 4-HBA (mg/mL)
b
R = 0.1 , p = 0.67
R = 0.25 , p = 0.14
e
R = −0.34 , p = 0.14
R = 0.34 , p = 0.04
h
R = −0.22 , p = 0.36
R = 0.24 , p = 0.16
Concentration of CGA (mg/mL)
c
R = −0.12 , p = 0.56
R = −0.085 , p = 0.72
f
R = −0.14 , p = 0.49
R = −0.1 , p = 0.67
i
R = −0.34 , p = 0.078
R = 0.47 , p = 0.038
Concentration of QUE (mg/mL)
Cladode length (cm)
Cladode width (cm)
Cladode thickness (cm)

Figure S6. The effect of cladode order counted from soil level or of the number of cladodes above a given cladode on the concentration of a, b – 4-hydroxybenzoic acid (4-HBA), b, e – chlorogenic acid (CGA), and f, c – quercetin (QUE) in the vegetative tissues of female and hermaphrodite cladodes. The probability of detection of 4-HBA and of QUE was lower in cladodes bearing more levels of daughter cladodes. *P* – probability of adjustment of the GLM model for a given sexual form

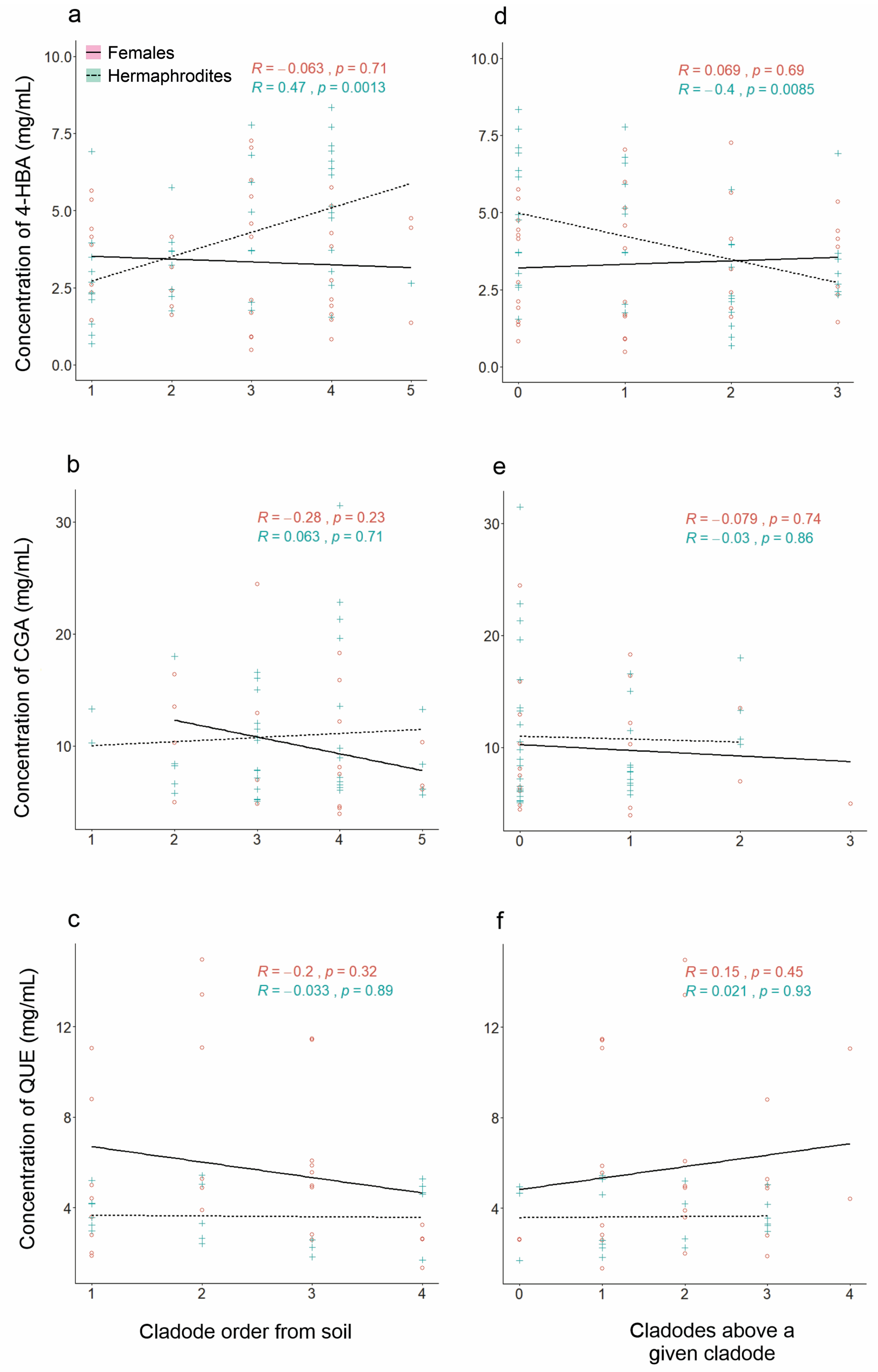

a
b
c
d
e
f
Females
Hermaphrodites
R = −0.063 , p = 0.71
R = 0.47 , p = 0.0013
R = 0.069 , p = 0.69
R = −0.4 , p = 0.0085
R = −0.28 , p = 0.23
R = 0.063 , p = 0.71
R = −0.079 , p = 0.74
R = −0.03 , p = 0.86
R = −0.2 , p = 0.32
R = −0.033 , p = 0.89
R = 0.15 , p = 0.45
R = 0.021 , p = 0.93
Concentration of 4-HBA (mg/mL)
Concentration of CGA (mg/mL)
Concentration of QUE (mg/mL)
Cladode order from soil
Cladodes above a given cladode

Supplementary Discussion S1

Do female individuals reveal a higher incidence/concentration of secondary metabolites than hermaphrodite individuals?

Do younger vegetative tissues reveal a higher incidence/concentration of SMs than older vegetative tissues?

Two secondary metabolites, 4-HBA and QUE showed dynamics during eight months. The general pattern of both metabolites was an increase of the proportion of cladodes bearing them at the beginning of the season and its decrease later on. The fact that the proportion of cladodes changed dynamically means that metabolites were either relocated among cladodes from different ages or cladodes stopped producing them. Independently of the proximate causes, this result is not concordant either with ODH or with RAH, for example, because the proportions of cladodes bearing two substances increased in October, when plants' tissues were older, than in previous months. The probability of detection of CGA did not fallow in statistically significant manner the predictions of any of the hypotheses mentioned: neither sexual forms nor season-specific cladode age differed in this trait. The lack of seasonal dynamics of CGA suggests either a lower mobility and/or a higher stability of this metabolite in the tissues of *O. robusta* and is parallel with the results of our field experiment [36].

The lack of significant dynamics of the concentration of 4-HBA for the entire model was an effect of the lack of these dynamics for female cladodes, since hermaphrodite cladodes presented differences in concentration of this metabolite among months. For example, in March and May, they were higher than in summer and autumn months.

An inverse relationship between the 4-HBA concentrations and the proportion of cladodes bearing it comparing both sexual forms might have been an outcome of either a trade-off, or of different defensive strategies displayed by the sexual forms. Hermaphrodite plants, on average, were less defended than female plants, since only a few of their cladodes contained this secondary metabolite, but those with its presence, contained it more concentrated. To the best of our knowledge, the energetic production cost of the 4-HBA in vivo is unknown so, we cannot conclude whether this inverse for both sexual forms concentration vs. probability of detection, is an expression of a trade-off, or of the "decision" of the plant of how to distribute this secondary metabolite: higher concentrations in few cladodes or lower, in more cladodes. As this secondary metabolite showed dynamics during eight-months study period and in another study of us we showed that 4-HBA can be transferred from apical to basal cladodes [36], the latter outcome is possible.

Contrary to the lack of significant dynamics of the proportion of cladodes bearing CGA, its average concentration in hermaphrodites in June was higher than in March, April, September and October. An increase of the concentration of this metabolite in some cladodes without an effect on their proportion bearing it, was an outcome of its low mobility between cladodes and its autonomous production by each cladode [36]. The concentration of CGA in cladodes did note fallow the predictions of both, ODH and RCH: it was lower at both, higher (spring) and lower habitat productivities (autumn; based on the values of the potential evapotranspiration). Also, it did not fallow the predictions of these hypotheses, because its dynamics was similar in both sexual forms.

The dynamics of the proportions of cladodes bearing 4-HBA and QUE are qualitatively similar in both sexual forms even when both metabolites responded in opposite way to the same meteorological factors. It means that other factors were on average more important for these dynamics than meteorological factors, for example, differential cost of plant tissues and thus, differential outcomes of optimal resources allocation to the production of theses metabolites and to other physiological processes.

The pattern of the concentrations of QUE reassembled the pattern of its distributions in cladodes. As QUE moves from apical to basal cladodes [36] , a trade-off between the concentration and the proportion of cladodes bearing it could have occurred, but it was not a phenomenon here: it was rather a “decision” of a plant to distribute this secondary metabolite in this way.

Table S1 online. Morphometric traits of *Opuntia robusta* from San Nicolas Tecoaco, Municipality of Singuilucan, Hidalgo State, Central Mexico, cladode order counted from soil, number of cladodes above a given cladode, date of sampling, spine colour (cladode age estimator) absorbance (surface below the absorbance curve), calibrations equations, and concentration of 4-hydroxybenzoic acid (4-HBA), quercetin (QUE), salicylic acid (SAL), and chlorogenic acid (CGA) obtained from the calibration equations for females and hermaphrodite cladodes. https://doi.org/10.7910/DVN/LERCFK/BI2OSB

Table S2 online. Results of structural equation models (SEM) for the relationship between meteorological variables and concentrations of three secondary metabolites or proportions of cladodes bearing them in female and hermaphrodite individuals of *Opuntia robusta* from San Nicolas Tecoaco, Municipality of Singuilucan, Hidalgo State,

Mexico 4-HBA - 4-hydroxybenzoic acid CGA - chlorogenic acid QUE - quercetin * - P < 0.05, ** - P < 0.01, and *** - P< 0.001. ** - P < 0.01 *** - P< 0.001. To determine the effects of the environmental variables on the concentration and presence/absence of secondary metabolites, we used the R software to formulate a structural equation model (SEM) in piecewiseSEM[30,37].

Meteorological data for Singuilucan Municipality, 2014 – Average or additive per-month data; Graphics: Histograms for average and additive variables; Graphics Per-Day – Graphics for sampling day; Coef Determ – Coefficients of determination and t-test probability for correlation coefficients; per-month average or additive variables Coef Determ_Per-Day – Coefficients of determination and t-test probability for correlation coefficients; averages or sums for additive variables for the sampling day; Correl. Per-Month vs. Per-Day – correlations coefficients, coefficients of determination, and t-test probability fore the relationship among per-month and per-sampling-day; data March-October – meteorological data for March through October 2014: lacking data that were adjusted, are marked with red fonts. Meteorological data obtained from: https://clima.inifap.gob.mx/lnmysr;

https://doi.org/10.7910/DVN/LERCFK/BI2OSB

Table S3 online. Descriptive statistics (exploratory analysis). We obtained the descriptive statistics for the proportion of cladodes bearing 4-Hydroxybenzoic acid (4-HBA), chlorogenic acid (CGA), and Quercetin (QUE), and we looked for the relationship between each dependent and independent variable: we used chi-squared test (sex, month), Cochran-Armitage (C-A) test (cladode age class, number of cladodes above a given

cladodes, cladode order above ground), and t-test (size estimators). With the C-A test we looked at how the probability of production of secondary metabolites depended on cladode age, cladode order and the number of cladodes the plant bore. We treated the variables of sex and month as categorical variables and estimated least square proportions for each cladode order. We considered the other variables to be continuous. We used the SAS statistical software [29]. https://doi.org/10.7910/DVN/LERCFK/BI2OSB

Table S3 online. Descriptive statistics (exploratory analysis). We obtained the descriptive statistics for the proportion of cladodes bearing 4-Hydroxybenzoic acid (4-HBA), chlorogenic acid (CGA), and Quercetin (QUE). We searched for the relationship between each dependent and independent variable: we used chi-squared test (sex, month), Cochran-Armitage (C-A) test (cladode age class, number of cladodes above a given cladodes, cladode order above ground), and t-test (size estimators). With the C-A test we looked at how the probability of production of secondary metabolites depended on cladode age, cladode order and the number of cladodes a given cladode bore. We treated the variables of sex and month as categorical variables and estimated least square proportions for each cladode order. We considered the other variables to be continuous. We used the SAS statistical software [29]. https://doi.org/10.7910/DVN/LERCFK/BI2OSB

Table S4 online. Results of GLMM model for the proportion of cladodes bearing 4-hydroxybenzoic acid (4-HBA), chlorogenic acid (CGA), and quercetin (QUE). We searched for the relationship between each dependent and independent variable. We applied the generalized linear mixed model (GLMM) with logit link function (ln(P/(1-P)),

where P – probability of detection of a given metabolite), binomial response distribution, maximum likelihood estimation technique, Newton-Raphson optimization algorithm, and Person Chi-Square/df fit criterion. We used GLIMMIX procedure of the SAS statistical software [29]. https://doi.org/10.7910/DVN/LERCFK/BI2OSB

Table S5 online. Model statistics for the best models and post-hoc contrasts. The effect of month, cladode size, cladode age cladode order from soil, number of daughter cladodes above a given cladode on the concentrations of 4-hydroxybenzoic acid (4-HBA), chlorogenic acid (CGA) and quercetin (QUE) in female and hermaphrodite cladodes of individuals of *Opuntia robusta* from Central Mexico.

To analyse how concentrations of the different substances were related to cladode length, width, thickness, months, age, cladodes order from soil, and cladodes above a given cladode, we used generalized linear models (GLMs) created in R (R Core Team, 2020). Since many concentrations were null, we analysed only the positive concentrations. The full model contained the interaction between each individual variable and sex. For each response variable, we used different error distribution and link functions that gave the best fit of the models. To analyse the effect of months and age on 4-HBA concentrations, we used the Gamma error distribution (GED) and the inverse link function. For the effect of months on CGA concentrations, we used GED and log link functions. For the effect of cladode age on CGA concentrations, we used GED and inverse link functions. For the effect of months on QUE concentration, we used GED and log link functions. For the effect of cladode age on QUE concentration, we used GED and inverse link function. For the models that related substances with cladode length, width and thickness, we used

different models: for 4-HBA concentrations we used a GED with square root link function. For CGA concentrations, we used GED and identity link function; for QUE concentrations, we used GED and inverse link function. To test the effect of cladodes order from soil on the concentrations of the three substances, we used GED and square root link function. To test the effect of the number of cladodes above a given cladode on the concentrations of the three substances, we used GED and inverse link function. We checked the goodness of fit of the linear models (GLM or GLMM) by plotting the standardized residuals against fitted values and by normal QQ-plots, and revised the assumptions of the homoscedasticity, proper distribution used, and independence. We chose the best GLM model used in this study according to the lowest AIC [31] following the recommendations of Zuur, et al.[32]. https://doi.org/10.7910/DVN/LERCFK/BI2OSB

Table S6 online. Least square linear regression functions for the relationships between the age-dependent proportions of cladodes bearing 4-hydroxybenzoic acid (4-HBA), chlorogenic acid (CGA), and quercetin (QUE) for cladodes arranged by age or by month of sapling. Cladode age classes corresponded to the colour of the spines in the fallowing manner: 1 – yellowish, 2 – yellow, white base, 3 – white yellowish, 4 – white, 5 – grayish, 6 – black, being "1" the youngest, and "6", the oldest age class. https://doi.org/10.7910/DVN/LERCFK/BI2OSB

Table S7 online. Least square linear regression functions for the relationship between *Opuntia robusta*'s cladode length and cladode order from soil or cladode age. Age estimator is based on the colour of the spines. Cladode age classes corresponded to the

colour of the spines in the fallowing manner: 1 – yellowish, 2 – yellow, white base, 3 – white yellowish, 4 – white, 5 – grayish, 6 – black, being "1" the youngest, and "6", the oldest age class. We compared slopes between sexual forms, for these relationships. https://doi.org/10.7910/DVN/LERCFK/BI2OSB

Table S8 online. Relationship between cladode length and cladode order from soil, and between cladode length and cladode age class, together with slope comparisons for female and hermaphrodite individuals. https://doi.org/10.7910/DVN/LERCFK/BI2OSB

Table S9 online. Least square linear regression functions for the relationship between cladode order from soil or nr of cladodes above, and cladode age estimator based on the colour of the spines. https://doi.org/10.7910/DVN/LERCFK/BI2OSB

References

1. Rzedowski, J. *Vegetación de México*. 1st digital edition edn, 504 (Comisión Nacional para el Conocimiento y Uso de la Biodiversidad, 2006).
2. Rzedowski, J. *Vegetación del Estado de San Luis Potosí*. (Faacultad de Ciencias, Universidad Nacional Autónoma de México 1961). https://books.google.com.mx/books?id=FCfAnQEACAAJ
3. del Castillo, R. F. *La selección natural de los sistemas de cruzamiento en Opuntia robusta* M.Sc. thesis, Colegio de Postgraduados, (1986).
4. García-Sánchez, R. *Patrones de polinización y fenología floral en poblaciones de Opuntia spp. en San Luis Potosí y Zacatecas* Bachelor's thesis thesis, Universidad Nacional Autónoma de México. México, D. F., (1984).
5. Sandoval-Molina, M. A. *et al.* First description of extrafloral nectaries in *Opuntia robusta* (Cactaceae): anatomy and ultrastructure. *PLoS One* **13**, e0200422 (2018). https://doi.org/10.1371/journal.pone.0200422
6. Boutakiout, A. *et al.* Effects of different harvesting seasons on antioxidant activity and phenolic content of prickly pear cladode juice. *Journal of the Saudi Society of Agricultural Sciences* **In press** (2016). https://doi.org/10.1016/j.jssas.2016.11.005

7. Saleem, M., Kim, H. J., Han, C. K., Jin, C. & Lee, Y. S. Secondary metabolites from *Opuntia ficus-indica* var. *saboten*. *Phytochemistry* **67**, 1390-1394 (2006). https://doi.org/10.1016/j.phytochem.2006.04.009
8. Guevara-Figueroa, T. *et al.* Proximate composition, phenolic acids, and flavonoids characterization of commercial and wild nopal (*Opuntia* spp.). *J. Food Comp. Anal.* **23**, 525-532 (2010). https://doi.org/10.1016/j.jfca.2009.12.003
9. Gonzalez-Ponce, H. A. *et al.* Hepatoprotective effect of *Opuntia robusta* and *Opuntia streptacantha* fruits against acetaminophen-induced acute liver damage. *Nutrients* **8**, 607 (2016). https://doi.org/10.3390/nu8100607
10. Janczur, M. K. *et al.* Chemical and physical defense traits in two sexual forms of *Opuntia robusta* in Central Eastern Mexico. *PLoS ONE* **9**, e89535 (2014). https://doi.org/10.1371/journal.pone.0089535
11. Erb, M. *et al.* Signal signature of aboveground-induced resistance upon belowground herbivory in maize. *Plant J.* **59**, 292-302 (2009). https://doi.org/10.1111/j.1365-313X.2009.03868.x
12. Erb, M. *et al.* Belowground ABA boosts aboveground production of DIMBOA and primes induction of chlorogenic acid in maize. *Plant Signal. Behav.* **4**, 639-641 (2014). https://doi.org/10.4161/psb.4.7.8973
13. Kumar, P., Ortiz, E. V., Garrido, E., Poveda, K. & Jander, G. Potato tuber herbivory increases resistance to aboveground lepidopteran herbivores. *Oecologia* **182**, 177-187 (2016). https://doi.org/10.1007/s00442-016-3633-2
14. Leiss, K. A., Maltese, F., Choi, Y. H., Verpoorte, R. & Klinkhamer, P. G. L. Identification of chlorogenic acid as a resistance factor for thrips in *Chrysanthemum*. *Plant. Physiol.* **150**, 1567-1575 (2009). https://doi.org/10.1104/pp.109.138131
15. Nuessly, G. S. *et al.* Resistance to *Spodoptera frugiperda* (Lepidoptera: Noctuidae) and *Euxesta stigmatias* (Diptera: Ulidiidae) in sweet corn derived from exogenous and endogenous genetic systems. *J. Econ. Entomol.* **100**, 1887-1895 (2007). https://doi.org/10.1603/0022-0493(2007)100[1887:RTSFLN]2.0.CO;2
16. Warabieda, W., Miszczak, A. & Olszak, R. W. The influence of methyl jasmonate (JA-Me) and B-glucosidase on induction of resistance mechanisms of strawberry against two-spotted spider mite (*Tetranychus urticae* Koch.). *Commun. Agric. Appl. Biol. Sci.* **70**, 829-836 (2005).
17. Mallikarjuna, N., Kranthi, K. R., Jadhav, D. R., Kranthi, S. & Chandra, S. Influence of foliar chemical compounds on the development of *Spodoptera litura* (Fab.) in interspecific derivatives of groundnut. *J. Appl. Entomol.* **128**, 321-328 (2004). https://doi.org/10.1111/j.1439-0418.2004.00834.x
18. Kliebenstein, D. J. Secondary metabolites and plant/environment interactions: a view through *Arabidopsis thaliana* tinged glasses. *Plant Cell Environ.* **27**, 675-684 (2004). https://doi.org/10.1111/j.1365-3040.2004.01180.x
19. Mikulic-Petkovsek, M., Usenik, V. & Stampar, F. The role of chlorogenic acid in the resistance of apples to apple scab (*Venturia inaequalis* (Cooke) G. Wind. Aderh.). *Zb. Bioteh. Fak. Univ. Ljublj. Kmet.* **81**, 233-242 (2003).
20. Smith-Becker, J. *et al.* Accumulation of salicylic acid and 4-hydroxybenzoic acid in phloem fluids of cucumber during systemic acquired resistance is

preceded by a transient increase in phenylalanine ammonia-lyase activity in petioles and stems. *Plant. Physiol.* **116**, 231-238 (1998). https://doi.org/10.1104/pp.116.1.231
21. Wuyts, N., Swennen, R. & De Waele, D. Effects of plant phenylpropanoid pathway products and selected terpenoids and alkaloids on the behaviour of the plant-parasitic nematodes *Radopholus similis*, *Pratylenchus penetrans* and *Meloidogyne incognita*. *Nematology* **8**, 89-101 (2006). https://doi.org/10.1163/156854106776179953
22. Padmavati, M., Sakthivel, N., Thara, K. V. & Reddy, A. R. Differential sensitivity of rice pathogens to growth inhibition by flavonoids. *Phytochemistry* **46**, 499-502 (1997). https://doi.org/10.1016/S0031-9422(97)00325-7
23. Parvez, M. M., Tomita-Yokotani, K., Fujii, Y., Konishi, T. & Iwashina, T. Effects of quercetin and its seven derivatives on the growth of *Arabidopsis thaliana* and *Neurospora crassa*. *Biochem. Syst. Ecol.* **32**, 631-635 (2004). https://doi.org/10.1016/j.bse.2003.12.002
24. Schweiger, R., Heise, A. M., Persicke, M. & Müller, C. Interactions between the jasmonic and salicylic acid pathway modulate the plant metabolome and affect herbivores of different feeding types. *Plant Cell Environ.* **37**, 1574-1585 (2014). https://doi.org/10.1111/pce.12257
25. Caarls, L., Pieterse, C. M. J. & Van Wees, S. C. M. How salicylic acid takes transcriptional control over jasmonic acid signaling. *Front. Plant. Sci.* **6**, 170 (2015). https://doi.org/10.3389/fpls.2015.00170
26. War, A. R., Paulraj, M. G., War, M. Y. & Ignacimuthu, S. Role of salicylic acid in induction of plant defense system in chickpea (Cicer arietinum L.). *Plant Signal. Behav.* **6**, 1787-1792 (2011). https://doi.org/10.4161/psb.6.11.17685
27. War, A. R. *et al.* Mechanisms of plant defense against insect herbivores. *Plant Signal. Behav.* **7**, 1306-1320 (2012). https://doi.org/10.4161/psb.21663
28. Sperandei, S. Understanding logistic regression analysis. *Biochem. Med. (Zagreb)* **24**, 12-18 (2014). https://doi.org/10.11613/BM.2014.003
29. SAS Institute Inc. SAS System for Windows v. 9.4 (SAS Institute Inc., 2017).
30. R Core Team. *R: A language and environment for statistical computing*. (R Foundation for Statistical Computing, 2020). www.R-project.org/
31. Akaike, H. Factor analysis and AIC. *Psychometrika* **52**, 317-332 (1987). https://doi.org/10.1007/BF02294359
32. Zuur, A. F., Ieno, E. N. & Elphick, C. S. A protocol for data exploration to avoid common statistical problems. *Methods Ecol. Evol.* **1**, 3-14 (2010). https://doi.org/10.1111/j.2041-210X.2009.00001.x
33. Janczur, M. K., González-Camarena, E. & Jenner, B. Dataset for Supplementary Table S2 for: Implications of the existence of separated sexual forms of *Opuntia robusta* for plant defense hypotheses, DRAFT VERSION. (Harvard Dataverse, 2020). https://doi.org/10.7910/DVN/LERCFK
34. Janczur, M. K., González-Camarena, E. & Jenner, B. Dataset for Implications of the existence of separated sexual forms of *Opuntia robusta* for plant defense hypotheses, V1. (Harvard Dataverse, 2020). https://doi.org/10.7910/DVN/LERCFK

35. Janczur, M. K., Sandoval-Molina, M. A. & González-Camarena, E. Dataset for Statistical analysis (GLMM) for: Implications of the existence of separated sexual forms of *Opuntia robusta* for plant defense hypotheses, V.1. (Harvard Dataverse, 2020). https://doi.org/10.7910/DVN/USSQ3F
36. León-Solano, H. J. *et al.* Resource allocation among cladodes of *Opuntia robusta* from the Central-Eastern Mexico. (2020). https://doi.org/10.21203/rs.3.rs-161086/v1
37. Lefcheck, J. S. piecewiseSEM: Piecewise structural equation modelling in r for ecology, evolution, and systematics. *Methods Ecol. Evol.* **7**, 573-579 (2016). https://doi.org/10.1111/2041-210X.12512
